# 9-Norlignans: Occurrence, Properties and Their Semisynthetic Preparation from Hydroxymatairesinol

**DOI:** 10.3390/molecules24020220

**Published:** 2019-01-09

**Authors:** Patrik Eklund, Jan-Erik Raitanen

**Affiliations:** Johan Gadolin Process Chemistry Centre, Laboratory of Organic Chemistry, Åbo Akademi University, Piispankatu 8, FIN-20500 Turku, Finland; jraitane@abo.fi

**Keywords:** lignans, norlignans, 9-norlignans, semisynthesis, hydroxymatairesinol, bioactivity

## Abstract

Lignans, neolignans, norlignans and norneolignans constitute a large class of phenolic natural compounds. 9-Norlignans, here defined to contain a β–β’ bond between the two phenylpropanoid units and to lack carbon number 9 from the parent lignan structure, are the most rarely occurring compounds within this class of natural compounds. We present here an overview of the structure, occurrence and biological activity of thirty-five 9-norlignans reported in the literature to date. In addition, we report the semisynthetic preparation of sixteen 9-norlignans using the natural lignan hydroxymatairesinol obtained from spruce knots, as starting material. 9-Norlignans are shown to exist in different species and to have various biological activities, and they may therefore serve as lead compounds for example for the development of anticancer agents. Hydroxymatairesinol is shown to be a readily available starting material for the preparation of norlignans of the imperanene, vitrofolal and noralashinol family.

## 1. Introduction

Many secondary metabolites, including lignans, flavonoids, and coumarins are formed from phenylpropanoids originating from the well-known shikimic acid pathway. The parent structures are usually further oxidized and arranged into various structures. Norlignans, a subclass of lignans lacking one or more carbon atoms, seem to be a rather unknown class of natural products. Compared to other phenylpropanoids their occurrence, biosynthetic pathway, and their properties are much less reported in the literature. Especially 9-norlignans (lacking carbon 9 from the parent lignan skeleton) with guaiacyl (3-methoxy-4-hydroxyphenyl) moieties have rarely been reported in the literature, although they would be expected to be common norlignans as guaiacyl lignans are the most abundant class of lignans. To the best of our knowledge, (+)-imperanene, vitrofolal E and F, noralashinol A and C are the only guaiacyl type 9-norlignans which have been reported as plant constituents [1,2,3,4]. The unnatural enantiomer, (−)-*R*-imperanene, dehydroxyimperanenes and dihydrodehydroxy-imperanenes have also been synthesized [5,6]. Normally, common substitution patterns are found in norlignans, but the more rare 2,4,5-trisubstituted phenyl moieties seem to be present in several 9-norlignans. In this paper we review the occurrence and properties of natural compounds belonging to the class of 9-norlignans, and in addition, we report the semisynthesis of 9-norlignans from the natural lignan hydroxymatairesinol.

### 1.1. Classification and Nomenclature of Norlignans

The term lignan is defined as two phenyl propane units coupled together by a β–β’ bond, and if the same structural units are coupled in any other ways the product is called a neolignan. If the coupling between the units contains an ether function (fundamental parent structures) the compound is called an oxyneolignan. According to the present recommendations by the International Union of Pure and Applied Chemistry (IUPAC), the prefix nor (modification of the fundamental parent structure) is used when the lignan, neolignan or oxyneolignan lack one or more carbon atoms [7]. However, in the literature there are today over 60 compounds named as norlignans although the major part of these are lacking the β–β’coupling, which is the definition for the fundamental parent structure named lignan. In our opinion most of these structures should be named as norneolignans or noroxyneolignans (prefix nor + fundamental parent structure). The choice of the numbering of the lost carbon is also somewhat confusing. Numbering of the modified carbon skeleton (in this case nor) should be performed so that the modification is expressed by the lowest locant (number, unprimed). However, the choice of locants for the removal of a carbon atom (nor) is preferred in the order: unprimed, higher number. Consequently, there is a conflict when assigning structures to 9 or 7-norlignans or norneolignans. Although the exact definition of different norlignans seems a bit confusing and vague, we here define a norlignan as a plant-derived compound consisting of two phenylpropane units coupled in the propane moiety with a β–β’ bond and missing one or more carbon atoms.

**Figure 1:**
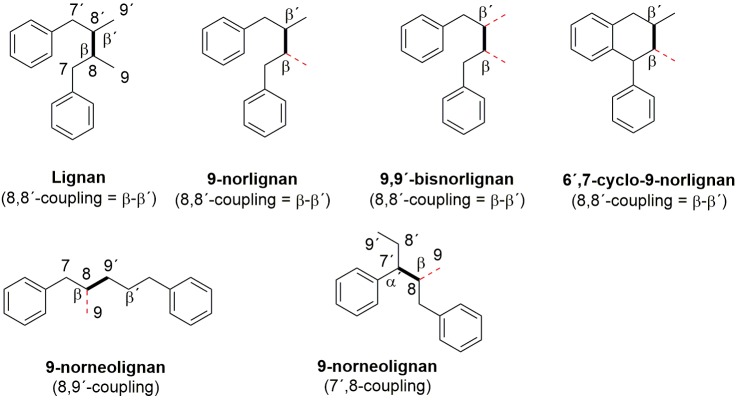
Fundamental structures for the lignan and different 9-norlignan and 9-norneolignan structures. Dotted (red lines) lines indicate the missing carbon-9 atom.

Moreover, we consider 9-norlignans to be derived from a parental lignan skeleton and thus to have a detectable β–β’ coupling and to lack one (or more, in the case of di(bis)norlignans) of the terminal carbon atoms (carbon 9). In Figure 1, the fundamental structures of different norlignan and norneolignan structures coupled in the propane moiety are displayed (oxyneolignans and others are excluded, dotted lines in red indicate the missing carbon-9, the bold lines indicate the coupling in the propane moiety).

### 1.2. 9-Norlignans

**Figure 2:**
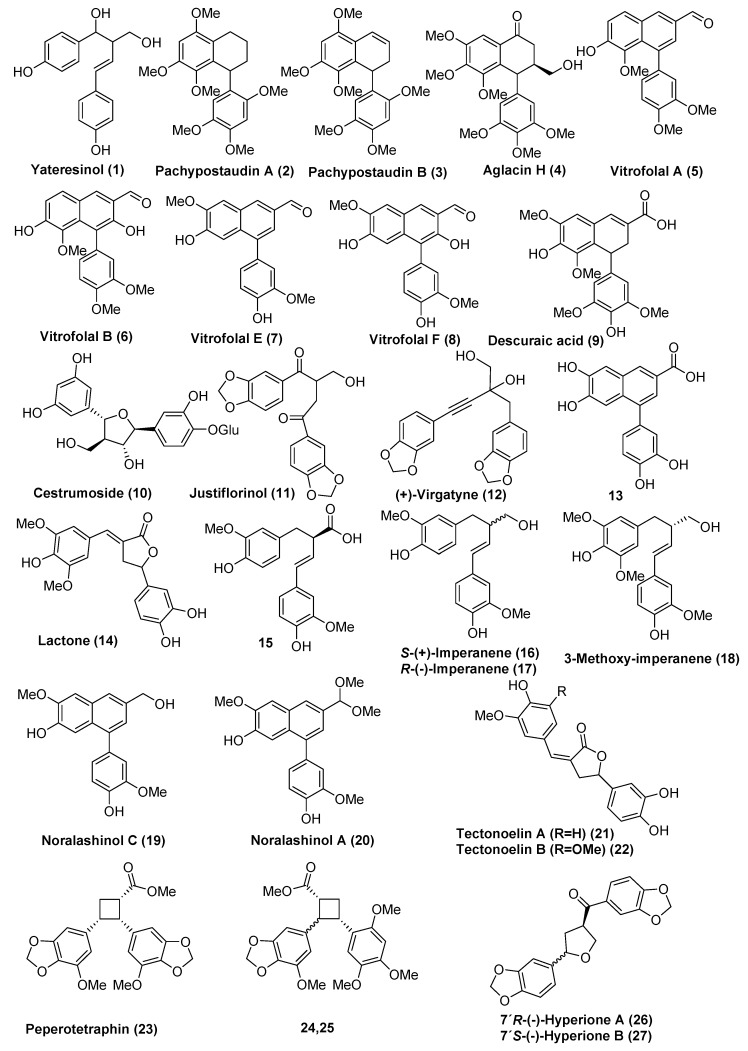
The structures of previously reported 9-norlignans with natural origin.

**Figure 3:**
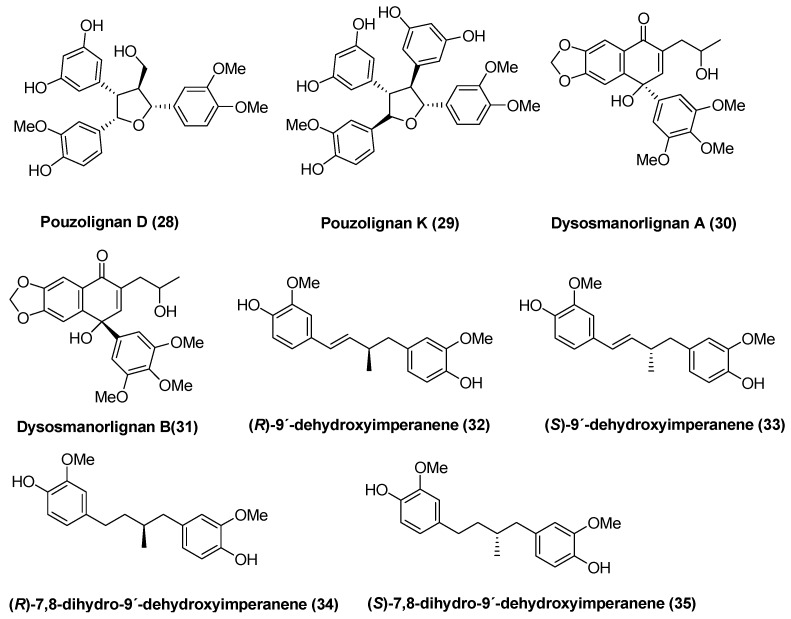
Structures of synthetic 9-norlignans and 9-norlignan related compounds.

According to the definition above, approximately 25 naturally occurring 9-norlignans can be found in the literature. These are yateresinol (**1**) [8], pachypostaudin A and B (**2**,**3**) [9], aglacin H (**4**) [10], vitrofolal A,B,E, and F (**5**,**6**,**7**,**8**) [2,11] descuraic acid (**9**) [12], cestrumoside (glycoside) (**10**) [13], (−)-justiflorinol (**11**) [14], (+)-virgatyne (**12**) [15], the arylnaphthalene derivative **13 [16]** and the lactone **14 [17]**, compound **15** [18], *S*-(+)-imperanene (**16**) [1], 3-methoxy-imperanene (**18**) [19], noralashinol A and C (**19**,**20**) [3,4], tectonoelin A and B (**21**,**22**) [20], peperotetraphin (**23**) [21] the related cyclobutane carboxylates **24** and **25 [22]** and hyperione A and B (**26**,**27**) [23] (Figure 2). In addition, some related 9-norlignans have been synthesized and some 9-norlignan like compounds have also been reported [6,24,25]. In Figure 3, the structures of compounds **28**–**35** are shown.

### 1.3. Occurrence and Biological Activity

Yateresinol (**1**) was first isolated from the heartwood of *Libocedrus yateensis* in 1979 by Ertman et al. [8]. It has also been found in *Cryptomerica japonica* D.Don and found to be part of the wood discoloring substances in sapwood [26]. Studies of butt-rot sugi wood suggested that the norlignans may be important for antifungal properties but no clear effect has been shown [27]. Yateresinol has been tested for cytotoxicity towards the human HL60 and Hepa G2 cancer cell lines [28] but was shown to be inactive with the half maximal inhibitory concentration (IC_50_) values >20 µg/mL. The two 9,9′-bisnorlignans **2** and **3** (pachypostaudin A and B) have been isolated from stem bark of *Pachypodanthium staudtii* Engl. et. Diels [9]. The same bisnorlignans have also been isolated from bark of the related species *Pachypodanthium confine* Engl. et Diels [29]. Both species are trees growing in the tropical zone of west and central Africa, and have been used in traditional African medicine. However, no biological activity of the bisnorlignans has been reported. Aglacin H (**4**) has been isolated from the bark of *Aglaia cordata* collected in Indonesia [10]. No other sources, and no chemical and biological properties for this norlignan have so far been reported. Norlignans from *Vitex* species, namely vitrofolal A, B, E, and F (compounds **5**–**8**) are norlignans of the arylnaphthalene type. These compounds were first isolated from *Vitex rotundifolia* [2,11]. Compounds **5**–**8** have later also been isolated from the seeds [30] and **7** from the roots of *Vitex negundo* [31,32]. Compounds **7** and **8** have also been isolated from the fruits of *Vitex cannabifolia* [33]. Vitrofolal E (**7**) was also recently reported in the stem and bark of *Syringa pinnatifolia* [2,34]. Vitrofolals have been shown to have various biological activities. Compound **7** was shown to have antibacterial activity against methicillin-resistant *Staphylococcus aureus* at concentrations above 64 µg/mL [2]. Compounds **7** and **8** have also been reported to have antioxidant activity by inhibition of lipid peroxidation using the ferric thiocyanate method, and by scavenging of the free radical 2,2-diphenyl-1-picrylhydrazyl (DPPH). In both assays **7** and **8** showed comparable or slightly higher antioxidative activity than α-tocopherol, l-cysteine and butylated hydroxyanisole (BHA) [33,35]. Vitrofolal E (**7**) has also been shown to have moderate activity in the in vitro cholinesterase inhibition assay [31] and to have tyrosinase inhibitory activity in the same µM-range compared to known inhibitors [32]. Vitrofolal F (**8**) has been shown to have α-chymotrypsin (serine protease) inhibitory effect and has thus been suggested as a specific natural inhibitor of this enzyme [36]. The aryldihydronaphthoic acid derivative, descuraic acid (**9**), was isolated from seeds of *Descurainia Sophia* (L.) Webb ex Prantl, an annual/biennial herb [12]. Despite the structural similarities with vitrofolals, no biological properties of this compound have been published to date, however it is used in traditional medicine to alleviate common cold symptoms and to prevent asthma and oedema. The 9-norlignan glycoside cestrumoside (**10**), was recently identified from methanol extract of *Cestrum diurnum* L. leaves [13]. No biological effects for this compound were reported, although several species of this plant have been used in traditional Chinese medicine, especially for treatment of burns and swellings. However, recently **10** was claimed to be a protein kinase C inhibitor in an animal feed additive [37]. A study of the cytotoxic effects of the arylnaphthalene lignans from Vietnamese Acanthaceae, *Justicia patentiflora* revealed the structure of justiflorinol (**11**). However, the cytotoxic evaluation showed that **11** does not have the same cytotoxic effect as structurally related lignans found in this plant species [14]. Justiflorinol has also been isolated from leaves of *Piper sanguineispicum* and tested for its antileishmanial activity and for cytotoxicity on MCF7 and Vero cells. No significant biological activity was found [38]. (+)-Virgatyne (**12**) has been found in whole plant extracts of the Taiwanese annual plant *Phyllantus virgatus* Frost. F. (Euphorbiaceae) [15]. This plant has traditionally been used to protect the liver and **12** was tested for anti-hepatitis B virus in a MS-G2 cell line, but was shown to be completely inactive [39]. 

Different liverwort species have been shown to contain dihydronaphthalene and naphthalene lignans and norlignans. The arylnaphthalene derivative **13** was first isolated from *Pellia epiphylla* and the structure was confirmed by chemical synthesis [16]. The same compound (**13**) was later found in *Jamesoniella autumnalis* [40], *Bazzania trilobata* [41], *Lepidozia reptans* [42], *Lepidozia incurvata*, *Chiloscyphus polyanthos* and *Jungermannia exsertifolia* ssp. *cordifolia*. In the last three species conjugates with malic acid, shikimic acid and α-L-rhamnose were also isolated [43], but so far no biological activity has been reported. 

The butyrolactone 9-norlignan (**14**) has been isolated from a methanol-water extract of leaves of *Cestrum parqui*, originally a South American shrub, but frequently occurring also in the Mediterranean region. Green cestrum (*Cestrum parqui*) has been shown to have phytotoxic effects and has been studied as a natural herbicide. Compound **14** showed phytotoxic effects on *Lactuca sativa* (lettuce) and *Lycopersicon esculentum* (tomato) by reducing the development of the plant [17]. 

Compound **15** was first identified in alkaline extraction (pulping) of Norway spruce (*Picea abies*) roots. Later it was also shown to be a degradation product of the abundant guaiacyl type dibenzylbutyrolactone lignan hydroxymatairesinol [18]. The degradation process has been studied in detail and **15** can be obtained by alkaline treatment of hydroxymatairesinol in nearly quantitative yields [5]. The carboxylic acid has not been reported as a constituent in other plants, but the corresponding alcohol ((−)-imperanene (**17**)), although the other enantiomer (+)-imperanene **16,** has been found in the rhizomes of *Imperata cylindrica*. (+)-Imperanene was shown to have platelet aggregation inhibitory effects and gave a complete inhibition, at 6 × 10^−4^ M concentration, of rabbit platelet aggregation induced by thrombin [1]. (+)-Imperanene, 3-methoxyimperanene (**18**) and their glucosides have also been detected in rum distillate wastewater. In the same report, these compounds were shown to be inhibitors of human tyrosinase activity (IC_50_ = 1.85 mM). (+)-Imperanene showed the highest activity, indicating that the guaiacyl (3-methoxy-4-hydroxyphenyl) substitution pattern is important for the activity [19]. Contrary to many other norlignans, imperanene has been a target for several synthetic works. Noralashinol C (**19**) and noralashinol A (**20**), structurally related to the arylnaphthalene norlignans vitrofolals, have been isolated from stem barks of *Syringa pinnatifolia*. Compound **20** seems to be the acetal of **7** (possibly formed by reaction with methanol). Compound **19** was inactive in a cytotoxicity assay using HepG2 hepatic cancer cells and **20** was inactive in the NO production inhibitory assay [3,4]. Tectonoelin A and B (**21**,**22**), and compound **14** have been isolated from the leaves of *Tectona grandis* and shown to have growth inhibition activity in the etiolated wheat coleoptiles bioassay test [20]. Interestingly, both green cestrum and teak leaves seem to contain these rare norlignans and display herbicidal effects. Cyclobutane-type norlignans peperoteraphin (**23**) and two related isomers (**24**,**25**) have been isolated from *Peperomia tetraphylla* (whole plant). Compounds **24** and **25** were tested for cytotoxicity in human liver cancer cell lines HepG2, human lung cancer cell lines A549, and human cervical cancer cell lines Hela. Moderate activity with IC_50_ values around 50 µM for all cell lines, were detected [21,22]. Hyperione A and B (**26**,**27**) have been isolated from *Hypericum chinense* (whole herb). *Hypericum* species are known for their antibacterial activity but compounds **26** and **27** showed no antibacterial activity [23]. The occurrence and biological activities of 9-norlignans are summarized in Table 1.

In addition to these compounds, some 9-norlignan-like compounds (although containing additional carbon atoms) have been isolated (Figure 3). Pouzolignan D (**28**) and K (**29**) were isolated from the aerial parts of *Pouzolzia zeylanica* (L.) Benn. var. *microphylla* (wedd.) W.T. Wang [24] and dysosmanorlignans A (**30**) and B (**31**) from the roots of *Dysosma versipellis* [25]. No biological activity has been reported for these compounds.

Several of these naturally occurring lignans have been the topic for total synthesis, however, the synthetic methods are excluded from this literature review. Some semisynthetic methods as well as the synthesis of interesting derivatives related to the presented norlignans will be discussed briefly. For example, the synthesized dehydroxy- and dihydrodehydroxyimperanene isomers have been evaluated for their plant growth regulatory effects, for larvicidal, antifungal, antibacterial and cytotoxic activity, and compared to dihydroguaiaretic acid isomers. In most studies compounds **32**–**35** were less or equally active as dihydroguaiaretic acids. In the cytotoxicity study, using HL-60 and HeLa cells, compound **35** showed the highest activity with IC_50_ values at approximately 6 µM, suggesting that norlignans may serve as lead compounds for anticancer agents [6].

As previously mentioned, imperanene derivatives can also be synthesized from the natural lignan hydroxymatairesinol and these compounds can be further transformed to vitrofolal and noralashinol type norlignans. To expand the substrate scope for investigation of 9-norlignans and to perform structure–activity relationship studies, we have undertaken the semisynthetic preparation of 15 different 9-norlignans with different functionalities using hydroxymatairesinol as starting material. In this strategy we chose to introduce flexibility or rigidity to the basic skeleton. We also varied the functionality at carbon-9 by the preparation of the carboxylic acid, the ester and the alcohol. In addition, the skeleton was also aromatized by oxidation and two different substitution patterns at the aromatic moieties were prepared. The semisynthesis of these compounds from hydroxymatairesinol is presented in detail below.

## 2. Results

### Semisynthesis of Norlignan Derivatives from Hydroxymatairesinol

**Scheme 1:**
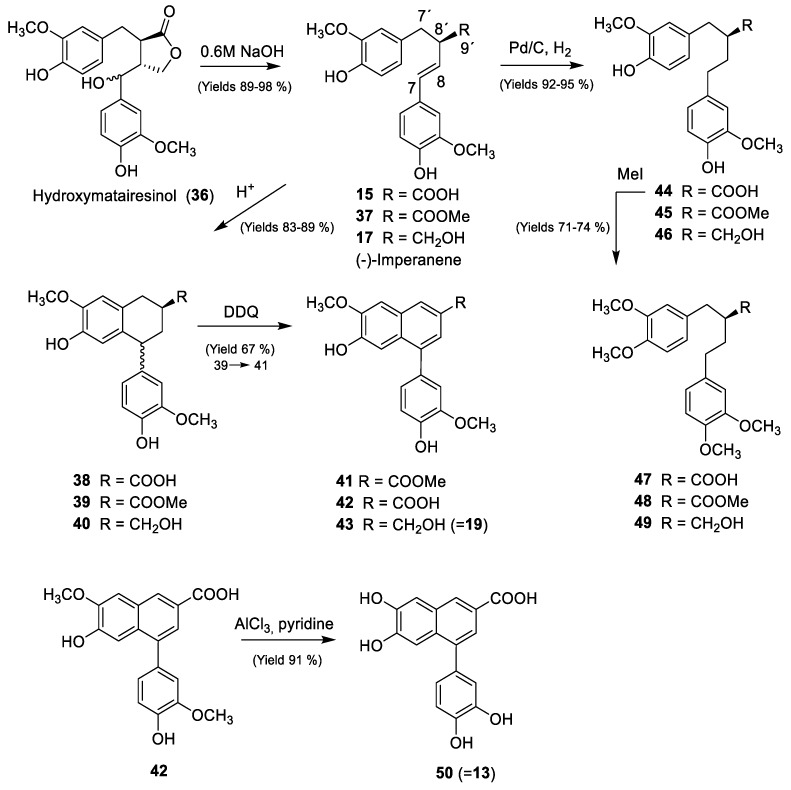
Preparation of various 9-norlignans from hydroxymatairesinol. This generalized scheme shows the fundamental chemical transformations. For more detailed information on the interconversions and transformations including reaction conditions, see the supplementary material.

The knots of Norway spruce (*Picea abies* (L.) H. Karst) have been shown to contain about 10% (*w*/*w*) of the dibenzylbutyrolactone hydroxymatairesinol (**36**, Scheme 1). Methods for the separation of knots and the subsequent isolation of hydroxymatairesinol were developed during the last decade. Today hydroxymatairesinol is isolated on kg-scale, which certainly makes it one of the most readily available lignans in pure form for further studies. In our previous studies, we have shown that hydroxymatairesinol is degraded to norlignan derivative **15** by strong alkali. This retro-aldol-type reaction probably involves a formation of a quinone methide intermediate and the loss of formaldehyde [5].

During degradation, two of the stereocentra of hydroxymatairesinol are destroyed, but the third retains the *R*-configuration without isomerization, yielding an optically pure product in excellent yields, usually over 95%. Further esterification and reduction of the obtained product (**37**), by lithium aluminium hydride (LAH) affords the 9-norlignan (−)-imperanene (**17**), as previously reported. However, the structure of **15** offers many possibilities for modification of the structure, which enabled the preparation of a set of derivatives for structure-activity relationship (SAR) studies of their biological effects (Scheme 1). The double bond was shown to be susceptible for protonation and Friedel–Crafts ring closing to yield two diastereomers of the 6’-7-cyclo-9-norlignan (aryltetralin-type) **38** in a 3:4 ratio. The two diastereomers were not separable by column chromatography. Further modification of the carboxylic acid to the ester **39** and the alcohol **40** was performed equally as in the case of **15** [5]. Oxidation of the cyclonorlignan **39** by 2,3-dichloro-5,6-dicyano-1,4-benzoquinone (DDQ) was utilized to aromatize the aliphatic ring to obtain the corresponding arylnaphthalene vitrofolal-type norlignan **41**. This structure was also transformed into the acid **42** and the alcohol **43** by hydrolysis and reduction, respectively. Oxidation of **38** or **40** by DDQ was proven to be unsuccessful. In the case of **38,** the structure was easily decarboxylated during the oxidation. The double bond of **15** was also reduced by catalytic hydrogenation in a batch reactor using ethanol, Pd/C and hydrogen at a slightly elevated pressure (2 bar) to give **44**. Again, the ester **45** and the alcohol **46** were prepared equally as for the other structures (Scheme 1). The permethylated derivative **48** was obtained by methylation with MeI in dry acetone and K_2_CO_3_. The acid **47** and the alcohol **49** were prepared accordingly. With these simple chemical modifications, we obtained a set of compounds (derivatives of imperanenes, vitrofolals and noralashinols) with some structural alterations. In this strategy we chose to introduce both flexibility, by reducing the double bond, and rigidity by introducing the aliphatic ring, at the basic skeleton of imperanene. The nonflexible cyclic structures with a half-boat structure **38**–**40** adopted a certain non-planar conformation, and were therefore aromatized to yield a planar structure (compounds **41**–**43**) resembling bioactive 9-norlignans of the vitrofolal and noralashinol family. Finally, we chose to prepare the carboxylic acid with an ionizable group, the ester containing the carbonyl function and the alcohol, for comparison of the effects of the functionality at C-9’. Some of the derivatives were also submitted to methylation of the free phenolic groups for comparison of the 3-methoxy-4-hydroxyphenyl and 3,4-dimethoxyphenyl moieties. The liverwort norlignan **13** was obtained from **42** by treatment with AlCl_3_ in pyridine. 

Compounds **15**, **44**, **48**, **37**, **38**, **40** and **41** have previously been evaluated as inhibitors of multidrug resistance protein 1 (MRP1)-mediated transport using human erythrocytes as model. This was part of a larger study on polyphenolic compounds comprising lignans, norlignans, stilbenes and flavonoids. Compound **15** was shown to be the most active one, with moderate activity at IC_50_ = 50 µM. The structure-activity relationship study showed that the carbonyl function at C-9’ is crucial for the activity. Furthermore, none of the norlignans showed detrimental effects up to 200 µM, indicating a large therapeutic width. These compounds could therefore be interesting as possible agents reversing multidrug resistance and to potentially sensitize cancer cells to anticancer drugs [44]. A similar set of compounds (**15**, **38**, **40**, **41, 44** and **48**) were tested for estrogen and antiestrogen activity in the yeast estrogen assay. Compounds **40**, **41** and **48** showed antiestrogenic effects, albeit at quite high concentrations (100–500 µM) [45]. Some of these norlignans were also studied for antimicrobial effects in comparison with the effective stilbene *trans*-pinosylvin (*trans*-3,5-dihydroxystilbene) and its monomethylated derivative (*trans*-3-hydroxy-5-methoxystilbene). Although some effects were observed, it was concluded that the norlignans were ineffective against *Salmonella infantis*, *Listeria monocytogenes* and *Candida tropicalis* [46].

## 3. Discussion and Conclusions

Norlignans of the C-9 type are an uncommon group of polyphenols in nature. Many of these compounds can be found in plants used in traditional Asian medicine, however published scientific data of their bioactivities is in general rather scarce. In most cases, their biological effects have been tested in a few assays mainly for antioxidant, cytotoxic and antimicrobial activities. Clearly, a more broad and systematic screening of bioactivity should be conducted to evaluate these compounds as potential bioactive agents. 9-Norlignans belonging to the vitrofolal-, noralashinol-, and imperanene families have however been shown to be bioactive and some of them could find potential in treatment of cancer. Furthermore, these 9-norlignans have been shown to inhibit specific enzymes, which warrants further investigations of these compounds. In our semisynthetic approach using hydroxymatairesinol as starting material for the preparation of 9-norlignans, we have developed simple chemical transformations to unsaturated, saturated, cyclic and aromatic structures closely resembling those of the vitrofolal, noralashinol and imperanene family. The unsaturated compounds **15**, **17** and **37** could also be seen as polyphenolic compounds with both *trans*-stilbene-like, and lignan-like structures, which could make them interesting as antimicrobial compounds. The cyclic structures were obtained in high yields by treatment with formic acid or trifluoroacetic acid. However, this Friedel–Crafts-type reaction can also be performed in aqueous conditions, using mineral acids. The aromatization was introduced by reaction with DDQ with moderate yields. DDQ was superior for this reaction due to its benzylic hydride abstraction properties, yielding dehydrogenation of the product, rather than oxidative polymerization. Hydrogenation of the double bond was facile, proceeding in high yields. To broaden the substrate scope of the semisynthetic 9-norlignans, the initial carboxylic acid (of compound **15**) was esterified and reduced by conventional methods, in relatively high yields. By these interconversions the carboxylic acid, the methyl ester and the alcohol derivatives were obtained for all the basic structures. Some structures were also methylated at the phenolic positions, which gave us 16 norlignans with different structures available for screening of biological activity. This semisynthetic route is not restricted to these compounds and it offers numerous additional chemical modifications and functional group interconversions. The biological testing of some selected semisynthetic 9-norlignans showed that none of these compounds were highly active. However, compound **15** showed moderate activity as an inhibitor of MRP1-mediated transport. The activity was decreased for all other derivatives, which indicated that the double bond and the carboxylic acid function were important for the activity. Due to the limited scope of biotesting for these semisynthetic 9-norlignans, no generalized results of their bioactivity or results on structure-activity relationships can be proposed, without further screening of biological activity. 

## 4. Materials and Methods

All commercially available chemicals were used as supplied by the manufacturers. Hydroxymatairesinol was isolated from Norway spruce (*Picea abies* (L.) Karst) knots by previously described methods [5]. Knots of Norway spruce were separated, ground and freeze-dried prior to extraction in a Soxhlet apparatus. The raw extract obtained with acetone-water (9:1 *v*/*v*), after the removal of lipophilic extractives with hexane was purified by flash chromatography (eluent CH_2_Cl_2_:EtOH 98:2 *v*/*v*) to yield hydroxymatairesinol.

GC analyses were performed on a standard gas chromatograph (Agilent Technologies, model 6850, Santa Clara, CA, USA) equipped with a HP-5 column and a flame ionization (FI) detector. The samples were silylated using hexamethyldisilazane-chlorotrimethylsilane in pyridine, prior to analyses. Gas chromatography-mass spectrometry (GCMS) analyses were performed essentially the same way (Agilent Technologies, model 7890A+5975C). High-resolution mass spectrometry (HRMS) were recorded using a Micro Q-TOF (Bruker, Billerica, MA, USA) with electrospray ionization (ESI) operated in positive mode or with a ZAB-Spec high-resolution MS-ESI instrument (Fisons Instruments, Ipswich, UK).

^1^H- and ^13^C-NMR spectra were recorded on an Avance instrument (Bruker) at 600.13 and 150.90 MHz, respectively. 2D NMR experiments (^1^H-^1^H COSY, HSQC, HMBC) were recorded using standard pulse sequences and chemical shifts are reported downfield from tetramethylsilane. Optical rotations were measured with a digital polarimeter model 241 (Perkin Elmer, Waltham, MA, USA) using a 1 dm, 1 mL cell.

The inhibition of multidrug resistance protein 1-mediated transport was studied by measurement of 2′,7′-bis-(carboxypropyl)-5(6)-carboxyfluorescein (BCPCF) efflux in human erythrocytes. Erythrocytes were loaded with 2′,7′-bis-(carboxypropyl)-5-(6)-carboxyfluorescein acetoxymethyl ester (BCPCF-AM) and incubated with or without the norlignans (10–200 mM). The extracellular BCPCF fluorescence intensity was then measured and the IC_50_ values for BCPCF efflux were determined from dose-response curves. The detailed experimental procedure and results have previously been published [44]. The estrogen and antiestrogen activity was determined using the yeast estrogen assay consisting of a transformed yeast strain (*Saccharomyces cerevisiae*). Cells were pre-cultured and diluted in a medium containing chlorophenol red-β-d-galactopyranoside. The suspensions with compounds were incubated in 96-well plates for three days before the absorbance at 540 nm was measured. Estrogenic or anti-estrogenic activity was calculated by subtraction of absorbance at 620 nm from that of 540 nm. Dose curves were plotted and the IC_50_ values were calculated. 17β-Estradiol was used as reference. A more detailed description has been published by Aehle et al. [45]. 

The antimicrobial activity tests were performed with *Listeria monocytogenes* L211, *Salmonella infantis* EELA72 and *Candida tropicalis* 4068 using an automated incubator and turbidity reader to monitor the microbial growth. The compounds were studied at equimolar concentrations (0.1–1 mM). The growth was calculated by subtraction of the turbidity of the test culture from that of the control and expressed as growth inhibition percentages. The detailed experimental procedure and results have previously been published [46].

## Figures and Tables

**Table 1:** 9-norlignans, their occurrence and biological activity.

Compound	Number	Occurrence	Bioactivity	References
Yateresinol	**1**	*Libocedrus yateensis, Cryptomerica japonica*	Antifungal	[8,26]
Pachypostaudin A	**2**	*Pachypodanthium staudtii, Pachypodanthium confine*		[9,29]
Pachypostaudin B	**3**			
Aglacin H	**4**	*Aglaia cordata*		[10]
Vitrofolal A	**5**	*Vitex rotundifolia, Vitex negundo, Vitex cannabifolia, Syringa pinnatifolia*	Antibacterial, antioxidant, cholinesterase inhibition, tyrosinase inhibitory effects, chymotrypsin inhibitory effects	[2,11,30,31,32,33,34,36]
Vitrofolal B	**6**			
Vitrofolal E	**7**			
Vitrofolal F	**8**			
Descuraic Acid	**9**	*Descurainia Sophia*		[12]
Cestrumoside	**10**	*Cestrum diurnum L.*		[13]
(−)-Justiflorinol	**11**	*Justicia patentiflora, Piper sanguineispicum*		[14,38]
(+)-Virgatyne	**12**	*Phyllantus virgatus*		[15]
Arylnaphthalene	**13**	*Pellia epiphylla, Jamesoniella autumnalis, Bazzania trilobata, Lepidozia reptans, Lepidozia incurvata, Chiloscyphus polyanthos, Jungermannia exsertifolia ssp. cordifolia*		[16,40,41,42,43]
Lactone	**14**	*Cestrum parqui*	Phytotoxic effects	[17]
Compound	**15**	*Picea abies*		[18]
(+)-Imperanene	**16**	*Imperata cylindrica, sugarcane rum distillate*	Platelet aggregation inhibitory effect, tyrosinase inhibitory effect	[1,19]
3-Methoxy-Imperanene	**18**	*Sugarcane rum distillate*	Tyrosinase inhibitory effect	[19]
Noralashinol C	**19**	*Syringa pinnatifolia*		[3,4]
Noralashinol A	**20**			
Tectonoelin A	**21**	*Tectona grandis*	Herbicidal activity	[20]
Tectonoelin B	**22**			
Peperoteraphin	**23**	*Peperomia tetraphylla*	Cytotoxic effect	[21,22]
Cyclobutane carboxylate	**24**			
Cyclobutane carboxylate	**25**			
Hyperione A	**26**	*Hypericum chinense*		[23]
Hyperione B	**27**			

## Supplementary Materials

The following are available online. Full experimental procedures for compounds **36**–**50**, including analytical data.
