# Supplementary material for: 9-Norlignans: Occurrence, Properties and Their Semisynthetic Preparation from Hydroxymatairesinol
Source: Molecules. 2019 Jan 9;24(2):220. doi: 10.3390/molecules24020220 (PMC6358742; doi:10.3390/molecules24020220)
Supplement: Supplementary file 1 [file molecules-24-00220-s001.pdf]

Supplementary Material

9-Norlignans: Occurrence, Properties and Their Semisynthetic Preparation from Hydroxymatairesinol

Patrik Eklund * and Jan-Erik Raitanen

Johan Gadolin Process Chemistry Centre, Laboratory of Organic Chemistry, Åbo Akademi University, Piispankatu 8, FIN-20500 Turku, Finland.

* Correspondence: paeklund@abo.fi; Tel.: +358-2-215-4720

Experimental

All commercially available chemicals were used as supplied by the manufacturers. Hydroxymatairesinol (**36**) (Scheme 1) was isolated from Norway spruce [*Picea abies* (L.) Karst] knots by the methods described previously [1,2]. Knots of Norway spruce were separated, ground and freeze-dried prior to extraction in a Soxhlet apparatus. The raw extract obtained with acetone-water (9:1 v/v), after the removal of lipophilic extractives with hexane, was purified by flash chromatography (eluent CH_2Cl_2:EtOH 98:2 v/v) to yield hydroxymatairesinol. Alternatively, knotwood material was extracted with ethanol and hydroxymatairesinol was precipitated by the addition of K-acetate to the ethanol extract. Compounds **15, 37** and **17** were prepared according to the previously published methods [2]. GC analyses were performed on a standard gas chromatograph equipped with a HP-5 column and a FI detector. The samples were silylated using hexamethyldisilazane-chlorotrimethylsilane in pyridine, prior to analyses. GCMS analyses were performed essentially the same way. HRMS were recorded using Bruker Micro Q-TOF with ESI (electrospray ionization) operated in positive mode or with Fisons ZAB-Spec high-resolution mass spectrometer. ^{1}H and ^{13}C spectra were recorded at 600.13 and 150.90 MHz, respectively. 2D experiments (COSY, HSQC, HMBC) were recorded using standard pulse sequences and chemical shifts are reported downfield from tetramethylsilane. Optical rotations were measured with a digital polarimeter using a 1 dm, 1 mL cell.

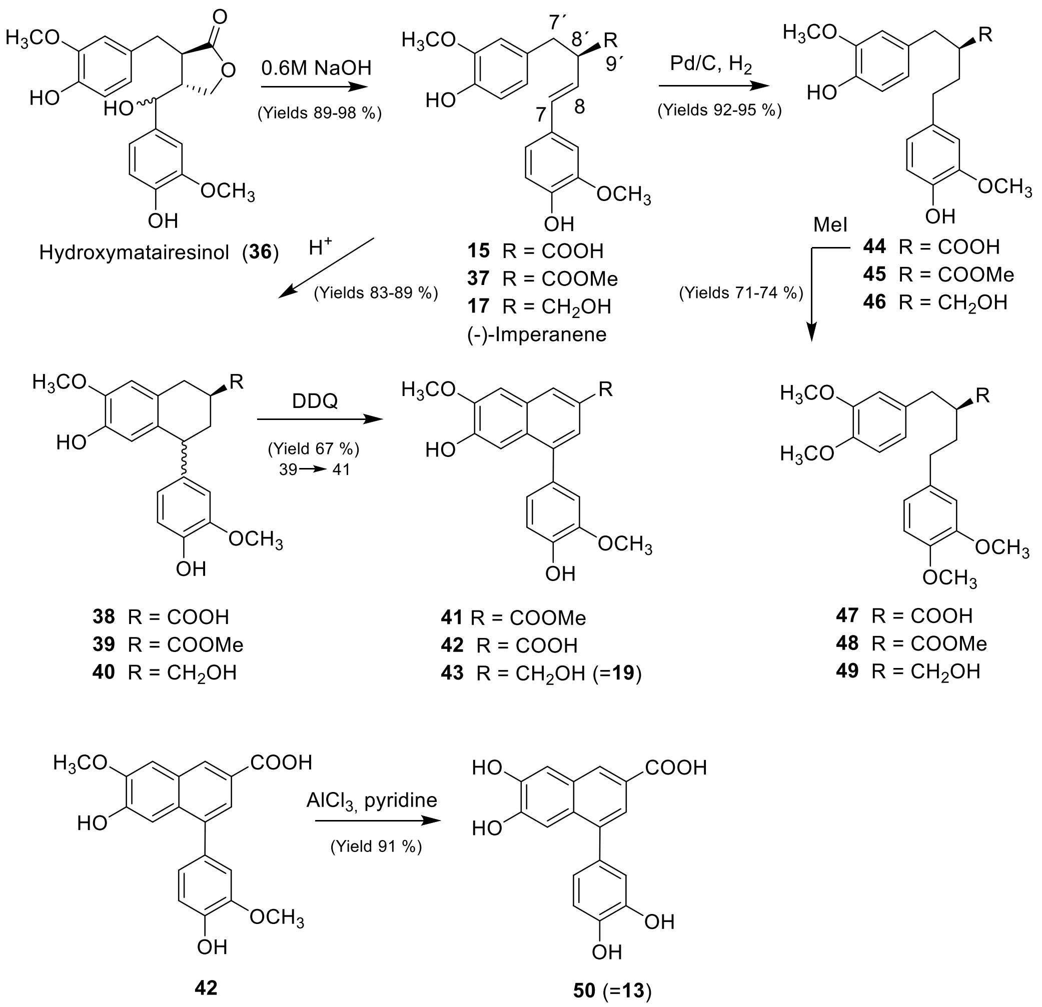

Scheme 1. Preparation of various 9-norlignans from hydroxymatairesinol, generalized overview.

(8′*S*,7*R*)-4,4′-dihydroxy-3,3′-dimethoxy-6′,7-cyclo-9-norlign-9′-oic acid (**38a**) and
(8′*S*,7*S*)-4,4′-dihydroxy-3,3′-dimethoxy-6′,7-cyclo-9-norlign-9′-oic acid (**38b**)

15 (1.0 g, 2.9 mmol) was dissolved in dichloromethane (50 ml) and trifluoroacetic acid (10 ml) was added. The mixture was stirred at room temperature for 3 h and then evaporated to a dark oil using a rotary evaporator. Dichloromethane (50 ml) and water (50 ml) were added to the residue, resulting in a dark green organic phase. The mixture was extracted with dichloromethane (4 x 50 ml), the organic phases were combined, washed with water (100 ml) and dried over Na_2SO_4. The solvent was removed using a rotary evaporator, yielding a brownish, fluffy product. Crystallization from chloroform gave a mixture of two diastereomers **38a** (7*R*) and **38b** (7*S*) in a 3:4 ratio (0.889 g, 89 %, purity 98 %, GCMS) after drying under vacuum.

HRMS (EI) *m/z* calculated for $C_{19}H_{20}O_6$: 344.1260 (M^+) obtained 344.1276.

38a EIMS (TMS-ethers) *m/z* 560 (M^+, 100 %), 545 (31), 442 (91), 411 (49), 247 (30), 222 (18), 209 (19), 73 (95).

^{1}H NMR (600 MHz, $CDCl_3$, 25 °C) d_H 2.14-2.18 (2H, m, H-8), 2.74-2.84 (1H, m, H-8′), 2.97-2.99 (2H, m, H-7′), 3.77 (3H, s, OCH_3′), 3.83 (3H, s, OCH_3), 4.12 (1H, dd, *J* = 5.0, 4.7 Hz, H-7) 6.37 (1H, s, H-5′), 6.38 (1H, dd, *J* =8.1, 2.0 Hz, H-6), 6.70 (1H, d, *J* = 8.1 Hz, H-5) 6.74, (1H, d, *J* = 2.1 Hz, H-2), 6.75 (1H, s, H-2′).
^{13}C NMR (151 MHz, $CDCl_3$, 25 °C) d_{13} 31.0 (C-7′), 33.7 (C-8), 35.3 (C-8′), 42.7 (C-7), 55.3-55.2 (OCH_3 × 2), 111.1 (C-2′), 112.4 (C-2), 114.0 (C-5), 116.3 (C-5′), 120.6 (C-6), 126.5 (C-6′), 130.2 (C-1′), 138.6 (C-1), 144.6 (C-4′), 145.1 (C-4), 146.3 (C-3′), 147.2 (C-3), 175.8 (C-9′).

38b EIMS (TMS-ethers) *m/z* 560 (M^+, 83 %), 545 (26), 442 (23), 411 (21), 304 (13), 242 (98), 222 (23), 73 (100).

^{1}H NMR (600 MHz, $CDCl_3$, 25 °C) d_H 1.85 (1H, q, *J* = 12.5, Hz, H-8a), 2.36 (1H, dddd, *J* = 12.5, 5.6, 2.0, 1.4 Hz, H-8b), 2,74-2,84 (1H, m, 8′), 2.90 (1H, dd, *J* = 16.3, 10.0 Hz, H-7′a), 3.02 (1H, dd, *J* = 16.3, 5.7, H-7′b), 3.78 (3H, s, -OCH_3′), 3.80 (3H, s, -OCH_3), 3.94 (1H, dd, *J* = 12.5, 5.6 Hz, H-7) 6.23 (1H, s, H-5′), 6.66 (1H, dd, *J* = 8.1, 2.0 Hz, H-6), 6.69 (1H, s, H-2′) 6.78 (1H, d, *J* = 8.1 Hz, H-5), 6.78 (1H, d, *J* = 2.0 Hz, H-2).
^{13}C NMR (151 MHz, $CDCl_3$, 25 °C) d_{13} 31.0 (C-7′), 36.8 (C-8), 40.4 (C-8′), 45.7 (C-7) 55.3 (OCH_3 × 2), 111.1 (C-2), 112.3 (C-2′), 115.1 (C-5), 115.2 (C-5′), 121.8 (C-6), 126.14 (C-6′), 132.0 (C-1′), 137.9 (C-1), 144.6 (C-4′), 145.1 (C-4), 145.9 (C-3′), 147.5 (C-3), 175.4 (C-9′).

(8′*S*,7*R*)-Methyl 4,4′-dihydroxy-3,3′-dimethoxy-6′,7-cyclo-9-norlign-9′-oate (**39a**) and **(8′*S*,7*S*)-Methyl 4,4′-dihydroxy-3,3′-dimethoxy-6′,7-cyclo-9-norlign-9′-oate 39b**)

Procedure 1 (from **37**)

37 (2.0 g, 5.6 mmol) was dissolved and stirred in TFA (22 ml) for 3 h at room temperature. The solvents were partially removed and the mixture was poured into water (100 ml) and extracted with dichloromethane (4 × 50 ml). The combined organic phase was washed with water (100 ml), dried over Na_2SO_4 and the solvent was removed under reduced pressure. The residue was purified by column chromatography (Kiselgel 60, Merck) using chloroform/methanol (98:2 v/v) as eluent to afford **39a** (7*R*) and **39b** (7*S*) in a 2:3 ratio (1.727 g, 86 %, purity 98 %, GCMS) after drying under vacuum.

Procedure 2 (from **38**)

A mixture of **38a** and **38b** (0.503 g, 1.46 mmol)) was dissolved in methanol (50 ml) and 0.2 ml H_2SO_4/MeOH (1:4 ratio) was added. The reaction was stirred for 17 h at 80 °C and then allowed to cool to room temperature. The reaction mixture was poured into saturated NaCl solution (80 ml) and was extracted with dichloromethane (4 × 30 ml). The combined organic phase was washed with water (100 ml), dried over Na_2SO_4 and the solvent was removed under reduced pressure. The residue was purified by column chromatography (Kiselgel 60, Merck) using chloroform/methanol (98:2 v/v) as eluent to afford **39a** (7*R*) and **39b** (7*S*) in a 1:3 ratio (0.442g, 85%) after drying under vacuum.

HRMS (EI) *m/z* calculated for $C_{20}H_{22}O_6$: 358.1416 (M^+) obtained 358.1415.

39a EIMS (TMS-ethers) *m/z* 502 (M^+, 100 %), 487 (14), 471 (12), 422 (17), 411 (18), 247 (21), 191 (13).

^{1}H NMR (600 MHz, $CDCl_3$, 25 °C) d_H 2.08-2.17 (2H, m, H-8), 2.71 (1H, dddd, J = 10.4, 10.2, 5.8, 3.5 Hz, H-8′), 2.87-2.91 (2H, m, H-7′), 3.59 (3H, s, OCH_3′), 3.77 (3H, s, OCH_3), 3.83 (3H, s, -

OCH_3), 4.05 (1H, dd, *J* = 4.7, 4.6 Hz, H-7), 6.35 (1H, dd, J = 8.2, 1.6 Hz, H-6), 6.43 (1H, s, H-5′), 6.49 (1H, d, *J* =1.6 Hz, H-2), 6.56 (1H, s, H-2′), 6.71 (1H, d, *J* = 8.2, H-5).
^{13}C NMR (151 MHz, $CDCl_3$, 25 °C) d_{13} 31.3 (C-7′), 34.4 (C-8), 35.5 (C-8′), 42.8 (C-7), 51.8, (OCH_3), 55.9 (Ar-OCH_3 × 2), 110.4 (C-2′), 111.1 (C-2), 113.9 (C-5), 115.6 (C-5′), 121.5 (C-6), 126.8 (C-6′), 130.1 (C-1′), 138.5 (C-1), 143.9 (C-4′), 144.2 (C-4), 145.4 (C-3′), 146.3 (C-3), 175.9 (C-9′).

39b EIMS (TMS-ethers) *m/z* 502 (M^+, 100 %), 487 (15), 306 (19), 247 (69), 228 (13), 217 (16) 191 (14).

^{1}H NMR (600 MHz, $CDCl_3$, 25 °C) d_H 1.82 (1H, ddd *J* = 12.6, 12.5, 12.4 Hz, H-8a), 2.34 (1H, ddd, 12.6, 3.0, 2.6 Hz, H-8b), 2,79 (1H, dddd J = 12.5, 12.0, 12.0, 4.8, 2.6 Hz, H-8′), 2.93-3.04 (2H, m, H-7′), 3.64 (3H, s, Ar-OCH_3′), 3.75 (3H, s, Ar-OCH_3′), 3.79 (3H, s, Ar-OCH_3), 3.86 (1H, dd J = 12.4, 3.0 Hz, H-7), 6.30 (1H, s, H-5′), 6.52 (1H, s, H-2′), 6.53 (1H, d, *J* = 1.7 Hz, H-2), 6.61, (1H, dd, *J* = 8.1, 1.7 Hz H-6), 6.77 (1H, d, *J* = 8.1 Hz, H-5).
^{13}C NMR (151 MHz, $CDCl_3$, 25 °C) d_{13} 32.0 (C-7′), 36.6 (C-8), 40.7 (C-8′), 45.9 (C-7) 51.8 (OCH_3), 55.9 (Ar-OCH_3 × 2), 110.5 (C-2), 110.8 (C-2′), 114.2 (C-5), 115.0 (C-5′), 121.6 (C-6), 126.6 (C-6′), 132.0 (C-1′), 137.8 (C-1), 143.8 (C-4′), 143.9 (C-4), 145.1 (C-3′), 146.5 (C-3), 175.6 (C-9′).

(8′*S*,7*R*)-4,4′-dihydroxy-3,3′-dimethoxy-6′,7-cyclo-9-norlign-9′-ol (**40a**) and
(8′*S*,7*S*)-4,4′-dihydroxy-3,3′-dimethoxy-6′,7-cyclo-9-norlign-9′-ol (**40b**)

A mixture of and **39a** and **39b** (0.195 g, 0.54 mmol) was dissolved in dry THF (20 ml) under an atmosphere of argon, LAH (0.1350 g, 6 eq.) was added portion wise. The mixture was stirred at 50 °C for 2 h. The reaction was stopped by pouring the mixture on saturated NaCl solution (50 ml). The pH was adjusted to 5 with HCl (10 % v/v) and the mixture was extracted with EtOAc (3 × 50 ml). The organic phases were combined, washed with saturated NaCl solution and dried over Na_2SO_4. The solvent was removed using a rotary evaporator and the residue was purified by column chromatography (Kiselgel 60, Merck) using EtOAc-Petroleum ether (60:40 v/v), yielding the desired product **40a** (7*R*) and **40b** (7*S*) after drying under vacuum (0.172 g, 96 %, purity 95 %, GCMS).

HRMS (EI) *m/z* calculated for $C_{19}H_{22}O_5$: 330.1467 (M^+) obtained 330.1456.

40a EIMS (TMS-ethers) *m/z* 546 (M^+, 100%), 456 (21), 441 (25), 428 (71), 261 (15), 247 (24), 209 (13).

^{1}H NMR (600 MHz, $CDCl_3$, 25 °C) d_H 1.82-1.87 (2H, m, H-8a), 1.91-1.92 (1H, m , H-8b), 1.99-2.05 (1H, m, H-8′), 2.50 (1H, dd, *J* = 16.3, 10.0 Hz, H-7′a), 2.91 (1H, dd, *J* = 16.3, 5.3 Hz, H-7′b), 3.56 (2H, d, *J* = 6.2 Hz, H-9′), 3.72 (3H, s, OCH_3′), 3.87 (3H, s, -OCH_3), 4.08 (1H, dd, *J* = 4.9, 4.5 Hz, H-7), 6.44 (1H, dd, *J* = 8.1, 1.8 Hz, H-6), 6.50 (1H, s, H-5′), 6.55 (1H, d, *J* =1.8 Hz, H-2), 6.62 (1H, s, H-2′), 6.76 (1H, d, *J* = 8.1Hz, H-5).
^{13}C NMR (151 MHz, $CDCl_3$, 25 °C) d_{13} 32.2 (C-7′), 32.3 (C-8′), 34.8 (C-8), 42.9 (C-7), 55.9 (OCH_3 × 2), 67.5 (C-9′) 110.7 (C-2′), 111.2 (C-2), 113.8 (C-5), 115.6 (C-5′), 121.5 (C-6), 127.9 (C-6′), 130.9 (C-1′), 139.4 (C-1), 142.8 (C-4′), 143.5 (C-4), 145.3 (C-3′), 146.2 (C-3).

40b EIMS (TMS-ethers) *m/z* 546 (M^+, 79 %), 456 (59), 428 (18), 261 (29), 247 (90), 230 (24), 209 (25).

^{1}H NMR (600 MHz, $CDCl_3$, 25 °C) d_H 1.49 (1H, m, H-8a) 2.06-2.13 (1H, m, H-8′), 2.14-2.19 (1H, m, H-8b), 2.60 (1H, dd, *J* = 16.3, 12.2 Hz, H-7′a), 2.85 (1H, dd, J = 16.3, 3.8 Hz, H-7′b), 3.63(1H, dd, *J* = 12.3, 6.3 Hz, H-9′), 3.80 (3H, s, -OCH_3′), 3.85 (3H, s, OCH_3), 3.89 (1H, dd *J* = 12.3, 5.6 Hz,

H-7), 6.36 (1H, s, H-5′), 6.59 (1H, s, H-2′), 6.61 (1H, d, *J* = 1.9 Hz, H-2), 6.67 (1H, dd, *J* = 8.0, 1.9 Hz, H-6), 6.83 (1H, d, *J* = 8.0 Hz, H-5).
^{13}C NMR (151 MHz, $CDCl_3$, 25 °C) d_{13} 33.0 (C-7′), 37.2 (C-8), 37.88 (C-8′), 46.1 (C-7), 55.9 (Ar-OCH_3 × 2), 68.0 (C-9′), 110.7 (C-2′), 110.8 (C-2), 114.2 (C-5), 115.0 (C-5′), 121.5 (C-6), 127,6 (C-6′), 133.0 (C-1′), 138.6 (C-1), 143.7 (C-4′), 144.1 (C-4), 144.9 (C-3), 146.5 (C-3′).

Methyl 4,4′-dihydroxy-3,3′-dimethoxy-9-nor-6′,7-cyclolign-7,7′-diene-9′-oate (41)

39 (0.301 g, 0.84 mmol) was dissolved in dichloromethane (100 ml) and freshly recrystallized DDQ (0.352 g, 1.85 eq.) was added. The mixture was stirred at room temperature for 2.5 h and then the mixture was filtered to remove $DDQH_2$. The solvent was partially removed and the precipitated DDQH was filtered again, the same procedure was repeated 3 times. Finally the residue was purified by column chromatography (EtOAc-Petroleum ether (50:50 v/v)) to yield **41** as a light yellow crystalline powder (200 mg, 67 %, purity 96 % GCMS) after drying under vacuum.

HRMS (EI) *m/z* calculated for $C_{20}H_{18}O_6$: 354.1103 (M^+) obtained 354.1098.

EIMS (TMS-ethers) *m/z* 498 (M^+, 100%), 469 (9), 468 (25), 438 (18), 379 (8), 226 (9), 73 (30), 59 (7).

^{1}H NMR (600 MHz, $(CD_3)_2CO$, 25 °C) d_H 3.90 (3H, s, Ar-OCH_3), 3.91 (3H, s, OCH_3), 4.04 (3H, s, Ar-OCH_3′), 6.92 (1H, dd, *J* = 2.0, 8.0 Hz, H-6), 6.98 (1H, d *J* = 8.0 Hz, H-5) 7.03 (1H, d, *J* = 2.0 Hz, H-2), 7.30 (1H, s, H-5′), 7.55 (1H, s, H-2′), 7.75 (1H, d, *J* = 1.5 Hz, H-8), 8.43 (1H, d, *J* = 1.5 Hz, H-7′).
^{13}C NMR (151 MHz, $(CD_3)_2CO$, 25 °C) d_{13} 52.2 (OCH_3), 56.3 (Ar-OCH_3′), 56.4 (Ar-OCH_3), 108.9 (C-2′), 109.1 (C-5′), 114.1 (C-2), 115.8 (C-5), 123.3 (C-6), 124.9 (C-8), 125.5 (C-8′), 129.3 (C-7′), 129.7 (C-1′), 131.6 (C-6′), 132.9 (C-1), 139.7 (C-7), 147.0 (C-4), 148.3 (C-3), 150.0-150.2 (C-3′, C-4′), 167.7 (C-9′).

4,4′-dihydroxy-3,3′-dimethoxy-9-nor-6′,7-cyclolign-7,7′-diene-9′-oic acid (42)

41 (0.2006 g, 0.56 mmol) was dissolved in NaOH (5M, 30 ml) and the solution was heated to 80 °C and stirred for 18 h. The reaction mixture was allowed to cool to room temperature and the pH was then adjusted to 1 with HCl (50 % v/v). EtOAc (50 ml) and water (50 ml) were added and the reaction mixture was extracted with EtOAc (5 × 50 ml), the organic phase was combined, washed with water (100 ml) and dried over Na_2SO_4. The solvent was removed using a rotary evaporator, yielding a light orange solid, which was dried under vacuum to obtain **42** (0.1693 g, 88 %, purity 98 %, GCMS).

HRMS (EI) *m/z* calculated for $C_{19}H_{16}O_6$: 340.0946 (M^+) obtained 340.0932.

EIMS (TMS-ethers) *m/z* 556 (M^+, 97 %), 541 (10), 526 (13), 378 (10), 263 (9), 248 (10), 241 (25), 73 (100)

^{1}H NMR (600 MHz, $(CD_3)_2SO$, 25 °C) d_H 3.81 (3H, s, Ar-OCH_3), 3.92 (3H, s, Ar-OCH_3′) 6.84 (1H, dd, *J* = 1.9, 8.0 Hz, H-6), 6.92 (1H, d *J* = 8.0 Hz, H-5) 6.98 (1H, d, *J* = 1.9 Hz, H-2), 7.26 (1H, s, H-5′), 7.54 (1H, s, H-2′), 7.62 (1H, d, *J* = 1.7 Hz, H-8), 8.37 (1H, d, *J* = 1.2 Hz, H-7′), 9.90 (1H, s, Ar-OH′), 9.92 (1H, s, Ar-OH).
^{13}C NMR (151 MHz, $(CD_3)_2SO$, 25 °C) d_{13} 55.6 (Ar-OCH_3), 55.7 (Ar-OCH_3′), 107.9 (C-5′), 108.5 (C-2′), 113.7 (C-2), 115.5 (C-5), 122.0 (C-6), 123.9 (C-8), 124.8 (C-8′), 128.2 (C-6′,C-7′), 129.9 (C-1′), 131.8 (C-1), 137.8 (C-7), 146.1 (C-4), 147.5 (C-3), 149.4 (C-3′), 149.4 (C-4′), 167.8 (C-9′).

4,4′-dihydroxy-3,3′-dimethoxy-9-nor-6′,7-cyclolign-7,7′-diene-9′-ol (43)

41 (0.3008 g, 0.85 mmol) was dissolved in dry THF (30 ml) and under an atmosphere of argon, LAH (0.1933 g, 6 eq.) was added portion wise. The mixture was stirred at 50 °C for 2h. The reaction was quenched by pouring the mixture on saturated NaCl solution (80 ml) and crushed ice. The pH was adjusted to 2 with HCl (10 % v/v) and the mixture was extracted with EtOAc (4 × 80 ml). The organic phases were combined, washed with saturated NaCl solution (80 ml) and dried over Na_2SO_4. The solvent was removed using a rotary evaporator and the product was chromatographed on a silica column using EtOAc-Petroleum ether (60:40 v/v), yielding **43** (0.2667 g, 96 %, purity 98 %, GCMS) as a light grey powder after drying under vacuum.

HRMS (EI) *m/z* calculated for $C_{19}H_{18}O_5$: 326.1154 (M^+) obtained 326.1156.

EIMS (TMS-ethers) *m/z* 542 (M^+, 100 %), 455 (12), 454 (33), 453 (15), 309 (28), 307 (18), 75 (66), 73 (72).

^{1}H NMR (600 MHz, $(CD_3)_2SO$, 25 °C) d_H 3.80 (3H, s, Ar-OCH_3), 3.89 (3H, s, Ar-OCH_3′), 4.59 (1H, d, *J* = 5.6 Hz, H-9′b), 4.60 (1H, t, *J* = 5.6 Hz, H-9′a), 5.17 (1H, d, *J* = 5.6 Hz, 9′-OH), 6.82 (1H, dd, *J* = 8.0, 2.0 Hz, H-6), 6.90 (1H, d *J* = 8.0 Hz, H-5′) 6.94 (1H, d, *J* = 1.9 Hz, H-2), 7.12 (1H, d, *J* = 1.7 Hz, H-8), 7.19 (1H, s, H-5′), 7.29 (1H, s, H-2′), 7.59 (1H, d, *J* = 0.7 Hz, H-7′), 9.08 (1H, s, Ar-OH), 9.36 (1H, s, Ar-OH′).
^{13}C NMR (151 MHz, $(CD_3)_2SO_2$, 25 °C) d_{13} 55.4 (Ar-OCH_3), 55.7 (Ar-OCH_3′), 63.2 (C-9′) 107.1 (C-2′), 107.9 (C-5′), 113.7 (C-2), 115.4 (C-5), 122.0 (C-6), 122.8 (C-7′), 124.0 (C-8), 126.3 (C-6′), 128.9 (C-1′), 131.9 (C-1), 136.8 (C-8′), 137.5 (C-7), 145.8 (C-4), 146.8 (C-4′), 147.4 (C-3), 148.9 (C-3′).

3,3′,4,4′-tetrahydroxy-9-nor-6′,7-cyclolign-7,7′-diene-9′-oic acid (50) (=**13**)

42 (0.1508 g, 0.44 mmol) was dissolved in pyridine (20 ml), cooled on an ice bath and under an atmosphere of argon, $AlCl_3$ (0.5919 g, 10 eq.) was added portion wise. The ice bath was removed and the reaction mixture was stirred at 60 °C for 20 h. Water (20 ml) was added to the reaction mixture and the pH was adjusted to 1 with HCl (50 % v/v). The mixture was extracted with EtOAc (5 × 30 ml). The organic phases were combined, washed with water (2×100 ml) and dried over Na_2SO_4. The solvent was removed by reduced pressure and the residue was dried under vacuum to give **50** (yield 0.1258 g, 91 %) as a light brown powder.

HRMS (EI) *m/z* calculated for $C_{17}H_{12}O_6Na$: 335.0531 (M^++Na) obtained 335.0521.

EIMS (TMS-ethers) *m/z* 672 (M^+, 82 %), 657 (7), 584 (6), 495 (10), 407 (5), 379 (8), 147 (3), 73 (100).

^{1}H NMR (600 MHz, $(CD_3)_2SO$, 25 °C) δ_H 6.70 (1H, dd, *J* = 2.1, 8.0 Hz, H-6), 6.82 (1H, d, *J* = 2.1 Hz, H-2), 6.87 (1H, d, *J* = 8.0 Hz, H-5), 7.24 (1H, s, H-5′), 7.31 (1H, s, H-2′), 7.50 (1H, d, *J* = 1.7 Hz, H-8), 8.21 (1H, d, *J* = 1.5 Hz, H-7′), 9.04 (1H, s, Ar-OH), 9.09 (1H, s, Ar-OH′), 9.68 (1H, s, Ar-OH′), 9.93 (1H, s, Ar-OH), 12.61 (1H, s, 9′-OH′).
^{13}C NMR (151 MHz, $(CD_3)_2SO$, 25 °C) δ_C 108.4 (C-5′), 111.9 (C-2′), 116.1 (C-5), 117.4 (C-2), 120.9 (C-6), 123.4 (C-8), 125.0 (C-8′), 127.9 (C-7′), 128.9 (C-6′), 129.8 (C-1′), 131.9 (C-1), 138.4 (C-7), 145.3 (C-3), 145.6 (C-4), 147.7 (C-3′), 149.6 (C-4′), 168.3 (C-9′).

(–)-(8′*R)*-4,4′-dihydroxy-3,3′-dimethoxy-9-norlign-9′-oic acid (44)

15 (8*R*′-dihydroxy-3,3′-dimethoxy-9′-norlign-7′-en-9-oic acid) **(**1.76 g, 5.11 mmol) was dissolved in ethanol (100 ml) and placed in a hydrogenation reactor. To the solution was added Pd/C (5 %) (0.18g) and the mixture was hydrogenated over hydrogen gas at a pressure of 2 bar for 5 h. The mixture was then filtrated and the solvent was removed under reduced pressure. The residue was re-dissolved and in dichloromethane, which gave precipitation upon cooling. The precipitation was filtered and dried under vacuum to yield **44** (1.53 g, 86 %, purity 98 %, GCMS).

$[a]^{D}_{20}$ = −17.1^{o} (c = 0.01 g/ml, Acetone).

HRMS (EI) *m*/*z* calculated for $C_{19}H_{22}O_6$: 346.1416 (M^+) obtained 346.1423.

EIMS (TMS-ethers) *m*/*z* 562 (M^+, 54%), 235 (9), 222 (16), 209 (100), 196 (73), 179 (31), 129 (10).

^{1}H NMR (600 MHz, $(CD_3)_2CO$, 25 °C) d_H 1.78 (1H, dddd, *J* = 13.5, 9.8, 6.7, 5.1 Hz, H-8a), 1.87-1.93 (1H, m, H-8b), 2.53 (1H, ddd, *J* = 13.8, 9.8, 6.7 Hz, H-7a), 2.63 (1H, ddd *J* = 13.8, 9.8, 5.5 Hz, H-7b), 2.65-2.69 (2H, m, H-8′), 2.72 (1H, dd, *J* = 13.5, 6.8 Hz, H-7′a, 2.90 (1H, dd, *J* = 13.5, 7.9 Hz, H-7′b), 3.80 (6H, s, 2 × OCH_3), 6.62 (1H, dd, *J* = 8.0, 1.8 Hz, H-6), 6.66 (1H, dd, *J* = 8.0, 1.8 Hz, H-6′), 6.72 (1H, d, *J* = 8.0 Hz, H-5′), 6.73 (1H, d, *J* = 8.0 Hz, H-5). 6.77 (1H, d, *J* = 1.8 Hz, H-2′), 6.81 (1H, d *J* = 1.8, H-2).
^{13}C NMR (151 MHz, $(CD_3)_2CO$, 25 °C) d_{13} 32.9 (C-7), 33.9 (C-8), 37.7 (C-7′), 46.7 (C-8′) 55.3 (OCH_3 × 2), 111.8 (C-2), 112.4 (C-2′), 114.6 (C-5′), 114.7 (C-5), 120.7 (C-6), 121,4 (C-6′), 130.9 (C-1′), 133.1 (C-1), 144.6 (C-4), 144.9 (C-4′), 147.1 (C-3), 147.2 (C-3′), 175.9 (C-9′).

(–)-(8′*R)*-Methyl 4,4′-dihydroxy-3,3′-dimethoxy-9-norlign-9′-oate (45)

44 (0.1504 g, 0.44 mmol) was dissolved in 50 ml MeOH and 0.1 ml MeOH:H_2SO_4 (4:1 ratio) was added. The solution was stirred at 50 °C for 20 h, cooled to room temperature and poured into saturated NaCl solution (50 ml). The mixture was extracted with dichloromethane (5 × 30 ml), the organic phases were combined, washed with water (100 ml) and dried over Na_2SO_4. The solvent was removed by evaporation to yield a chewy oil of **45** (0.1425 g, 91 %, purity 97 %, GCMS) after drying under vacuum.

$[a]^{D}_{20}$ = −12.1^{o} (c = 0.01 g/ml, EtOH).

HRMS (EI) *m*/*z* calculated for $C_{20}H_{24}O_6$: 360.1572 (M^+) och obtained 360.1569.

EIMS (TMS-ethers) *m*/*z* 504 (M^+, 75 %), 222 (6), 209 (100), 196 (11), 193 (10), 179 (55), 149 (8), 73 (51).

^{1}H NMR (600 MHz, $CDCl_3$, 25 °C) δ_H 1.74 (1H, m, H-8b), 1.94 (1H, m, H-8a), 2.48 (1H, ddd, *J* = 6.9, 9.4, 14.0 Hz, H-7b), 2.57 (1H, ddd, *J* = 5.6, 9.7, 14.1 Hz, H-7a), 2.64 (1H, m, H-8′), 2.68 (1H, dd, *J* = 7.0, 13.1 Hz, H-7′a), 2.89 (1H, dd, *J* = 7.7, 13.1 Hz, H-7′b), 3.63 (1H, s, 9′-OMe′), 3.83 (1H, s, Ar-OMe), 3.84 (Ar-OMe′), 5.49 (1H, s, Ar-OH), 5.51 (1H, s, Ar-OH′), 6.60 (1H, d, *J* = 1.9 Hz, H-2), 6.61 (1H, d, *J* = 1.9 Hz, H-2′), 6.62 (1H, dd, *J* = 2.0, 8.0 Hz, H-6), 6.63 (1H, dd, *J* = 8.0, 2.0 Hz, H-6′), 6.80 (1H, d, *J* = 8.0 Hz, H-5), 6.81 (1H, d, *J* = 8.0 Hz, H-5′).
^{13}C NMR (151 MHz, $(CD_3)_2SO_2$, 25 °C) δ_C 33.4 (C-7), 33.8 (C-8′), 38.4 (C-7′), 47.3 (C-8′), 51.6 (C-9′-OMe′), 55.9 (Ar-OMe, Ar-OMe′), 111.1 (C-2′), 111.4 (C-2), 114.2 (C-6), 114.3 (C-5), 121.1 (C-5′), 121.7 (C-6′), 131.2 (C-1′), 133.4 (C-1), 143.8 (C-4), 144.2 (C-4′), 146.5 (C-3′, C-3), 176.2 (C-9′).

(–)-(8′*R*)-4,4′-dihydroxy-3,3′-dimethoxy-9-norlign-9′-ol (46)

A mixture of a **44**/**45** (11:89 ratio, 0.894 g) was dissolved in dry THF (80 ml) and under an atmosphere of argon, LAH (0.5649 g, 6 eq.) was added portion wise. The mixture was stirred at 50 °C for 2.5 h. The reaction was stopped by pouring the mixture on saturated NaCl solution (100 ml) and crushed ice. The pH value was adjusted to 2 with HCl (10 % v/v) and the mixture was extracted with EtOAc (4 × 80 ml). The organic phases were combined, washed with saturated NaCl solution (150 ml) and dried over Na_2SO_4. The solvent was removed using a rotary evaporator to yield an oil (0.8273 g). The oil was chromatographed on a silica column using EtOAc-Petroleum ether (70:30 v/v) yielding **46** as an oil (0.6678 g, 81 %, purity 98 %, GCMS) after drying under vacuum.

$[a]^{D}_{20} = -2.6^{o}$ (c = 0.008 g/ml, EtOH).

HRMS (EI) *m/z* calculated for $C_{19}H_{24}O_5$: 332.1623 (M^+) obtained 332.1623.

EIMS (TMS-ethers) *m/z* 548 (M^+, 25 %), 249 (19), 236 (13), 210 (48), 209 (100), 180 (14), 179 (42). 73 (61).

^{1}H NMR (600 MHz, ($CDCl_3$, 25 °C) d_H 1.59 (1H, m, H-8b), 1.67 (1H, m, H-8a), 1.79 (1H, m, H-8′), 2.56 (1H, m, H-7b), 2.59 (1H, m, H-7a), 2.60 (1H, m, H-7′b), 2.62 (1H, m, H-7′a), 3.56 (1H, d, *J* = 5.0, 12.3 Hz, H-9′b), 3.57 (1H, d, *J* = 5.7, 12.3 Hz, H-9′a), 3.82 (6H, s, Ar-OCH_3, Ar- OCH_3′), 5.28 (1H, s, 9′OH), 5.57 (1H, s, Ar-OH), 5.59 (Ar-OH′), 6.60 (1H, d, *J* = 1.8 Hz, H-2), 6.62 (1H, *J* = 1.8 Hz, H-2′), 6.64 (1H, dd, *J* = 1.8, 8.0 Hz, H-6), 6.65 (1H, dd, *J* = 1.8, 8.0 Hz, H-6′), 6.80 (1H, d, *J* = 8.0 Hz, H-5). 6.81 (1H, d, *J* =8.0 Hz, H-5′).
^{13}C NMR (151 MHz, ($CDCl_3$, 25 °C) d_{13} 32.6 (C-8), 33.0 (C-7), 37.4 (C-7′), 42.1 (C-8′), 55.9 (Ar-OCH_3), 56.0 (Ar-OCH_3′), 65.0 (C-9′), 110.9 (C-2), 111.7 (C-2′), 114.3 (C-5′, C-5), 120.9 (C-6), 121.9 (C-6′), 132.5 (C-1′), 134.4 (C-1), 143.7 (C-4), 143.9 (C-4′), 146.5 (C-3′), 146.5 (C-3).

(–)-(8′*R*)-Methyl 3,3′,4,4′-tetramethoxy-9-norlign-9′-oate (48)

44 (0.964 g, 2.8 mmol) was dissolved in dry acetone (40 ml) and K_2CO_3 (3.09 g, 8 eq.) was added. To the mixture was then MeI (4.74 g, 12 eq.) added dropwise during 10 minutes. The mixture was refluxed for 42 h. The solvent was partially removed under reduced pressure and the (K_2CO_3) precipitation was removed by filtration. The residue was extracted with dichloromethane (3 × 30 ml) and water (25 ml). The organic phase was separated, dried over Na_2SO_4 and the solvent removed under reduced pressure. The residue was re-dissolved in ethyl acetate:dichloromethane (1:3 ratio), which gave a precipitation upon cooling. The precipitation was separated and dried under vacuum to yield **48** (0.764 g, 71 %, purity 98 %, GCMS) as a yellow powder.

$[a]^{D}_{20} = -12.2^{o}$ (c = 0.01g/ml, Acetone).

HRMS (EI) *m/z* calculated for $C_{22}H_{28}O_6Na$: 411.1783 (M^++Na) obtained 411.1780.

EIMS *m/z* 388 (M^+, 28 %), 205 (3), 177 (4), 164 (10), 151 (100), 138 (26), 107 (9).

^{1}H NMR (600 MHz, $(CD_3)_2CO$, 25 °C) d_H 1.79 (1H, dddd, *J* = 13.7, 9.7, 6.8, 5.0 Hz, H-8a), 1.90 (1H, dddd J = 13.7, 9.2, 6.0, 5.7 Hz, H-8b), 2,52 (1H, ddd, *J* = 13.8, 9.5, 6.8 Hz, H-7a), 2.59 (1H, ddd, *J* = 13.8, 9.7, 5.7 Hz, H-7b), 2.69 (1H, dddd, *J* = 8.8, 8.4, 6.4, 5.0 H-8′), 2.75 (1H, dd, *J* = 13.5, 6.4 Hz, H-7′a), 2.87 (1H, dd, *J* = 13.5, 8.4. Hz, H-7′b), 3.60 (3H, s, 9′-OCH_3), 3.75-376 (12H, s, Ar-

OCH_3) 6.67 (1H, dd, *J* = 8.2, 2.1 Hz, H-6), 6.68 (1H, dd, *J* = 2.2, 8.2 Hz, H-6′) 6.75 (1H, d, *J* = 2.2 Hz, H-2), 6.76 (1H, d, *J* = 2.2 Hz, H-2′), 6.81 (1H, d, *J* = 8.1 Hz, H-5), 6.83 (1H, d, *J* = 8.1Hz, H-5′).
^{13}C NMR (151 MHz, $(CD_3)_2CO$, 25 °C) d13 33.6 (C-7), 34.6 (C-8), 38.6 (C-7′), 47.8 (C-8′) 51.6 (OCH_3), 55.9 (Ar-OCH_3′ × 2), 56.0 (Ar-OCH_3 × 2) 112.7 (C-2), 112.9 (C-2′), 113.3 (C-5′), 113.6 (C-5), 121.1 (C-6), 121.7 (C-6′), 132.8 (C-1′), 135.1 (C-1), 148.7 (C-3), 148.9 (C-3′), 150.2 (C-4), 150.3 (C-4′), 176.1 (C-9′).

(−)-(8′*R*)- 3,3′,4,4′-tetramethoxy-9-norlign-9′-oic acid (47)

48 (0.300 g, 0.77 mmol) was dissolved in NaOH (5M, 40 ml) and the solution was heated to 80 °C and stirred for 18 h. The reaction mixture was allowed to cool to room temperature and the pH was then adjusted to 1 with HCl (50 % v/v). EtOAc (50 ml) and water (50 ml) was added and the reaction mixture was extracted with EtOAc (4 × 50 ml), the organic phases were combined, washed with water (100 ml) and dried over Na_2SO_4. The solvent was removed using a rotary evaporator, yielding an oil. According to GC-MS analysis, there was still starting material. The oil was added NaOH (5M, 40 ml) and stirred at 80 °C for 2.5 days. The pH value of the reaction mixture was adjusted to 1 with HCl (50 % v/v) and dichloromethane (50 ml) and water (50 ml) was added. The reaction mixture was extracted with dichloromethane (4 × 50 ml), the organic phases were combined and washed with water (150 ml) and dried over Na_2SO_4. The solvent was removed using a rotary evaporator, yielding and oil. The residue was re-dissolved in dichloromethane:diethylether (3:1 ratio), which gave a precipitation upon cooling. After a few days **47** (0.2245 g, 78 %, purity 98 %, GCMS) was obtained after drying under vacuum.

$[a]^{D}_{20}$ = −15.2° (c = 0.01 g/ml, EtOH).

HRMS (EI) *m/z* calculated for $C_{21}H_{26}O_6$: 374.1729 (M^+) obtained 374.1729.

EIMS (TMS-ethers) *m/z* 446 (M^+, 41%), 192 (11), 177 (10), 164 (23), 151 (94), 138 (100) 107 (10), 73 (13).

^{1}H NMR (600 MHz, $CDCl_3$, 25 °C) d_H 1.72 (1H, dddd, *J* = 4.6, 7.0, 9.9, 14.3 Hz, H-8a), 1.90 (1H, dddd *J* = 5.4, 9.2, 8.5, 14.3 Hz, H-8a), 2.48 (1H, ddd, *J* =7.0, 9.2, 14.2 Hz, H-7b), 2.60 (1H, m, H-7a), 2.63 (1H, m, H-8′), 2.66 (1H, dd, *J* =7.2, 13.4 Hz, H-7′b), 2.89 (1H, dd, *J* =7.3, 13.4, H-7′a), 3.75 (3H, s, Ar-OCH_3′), 3.77 (3H, s, Ar-OCH_3), 3.78 (6H, s, Ar-OCH_3, Ar-OCH_3′), 6.58 (1H, d, *J* = 2.0 Hz, H-2), 6.59 (1H, d, *J* = 2.0 Hz, H-2′), 6.61 (1H, dd, *J* = 2.0, 8.1 Hz, H-6), 6.62 (1H, d, *J* = 2.0, 8.1 Hz, H-6′), 6.69 (1H, *J* = 8.1 Hz, H-5′), 6.70 (1H, d, *J* = 8.1Hz, H-5).
^{13}C NMR (151 MHz, $CDCl_3$, 25 °C) d13 33.1 (C-7), 33.4 (C-8), 37.8 (C-7′), 46.8 (C-8′), 55.9 (Ar-OCH_3) 55.9 (Ar-OCH_3′), 56.0 (Ar-OCH_3), 56.0 (Ar-OCH_3′), 111.2 (C-5′), 111.3 (C-5), 111.8 (C-2), 112.1 (C-2′), 120.4 (C-6), 121.0 (C-6′), 131.4 (C-1′), 133.9 (C-1), 147.4 (C-4), 147.8 (C-4′), 148.9 (C-3, C-3′).

(−)-(8′*R*)- 3,3′,4,4′-tetramethoxy-9-norlign-9′-ol (49)

48 (0.2504 g, 0.64 mmol) was dissolved in dry THF (20 ml). Under an atmosphere of argon, LAH (0.1580 g, 6 eq.) was added portion wise. The reaction mixture was stirred at 50 °C for 2 h. The reaction was stopped by pouring the mixture on saturated NaCl solution (60 ml) with crushed ice, the pH was adjusted to 2 with HCl (10 % v/v) and extracted with EtOAc (4 × 80 ml). The organic phases were combined, washed with saturated NaCl solution (80 ml) and dried over Na_2SO_4. The solvent was removed using a rotary evaporator and the residue was dried under vacuum to give **49** (0.225 g, 97 % purity 96 %, GCMS) as a pale oil.

$[a]^{D}_{20}$ = −15.8° (c = 0.01 g/ml, EtOH).

HRMS (EI) *m/z* calculated for $C_{21}H_{28}O_5$: 360.1937 (M^+) obtained 360.1935.

EIMS (TMS-ethers) *m/z* 432 (M^+, 31%), 191 (21), 177 (18), 164 (16), 152 (55) 151 (100), 107 (9), 73 (14).

^{1}H NMR (600 MHz, $CDCl_3$, 25 °C) d_H 1.56 (1H, m, H-8b), 1.64 (1H, m, H-8a), 1.75 (1H, m, H-8′), 2.51 (1H, m, H-7b), 2.54 (1H, m, H-7a), 2.55 (1H, m, H-7′b), 2.56 (1H, m, H-7′a), 3.49 (1H, dd, *J* = 5.0, 10.8 Hz, H-9′b), 3.52 (1H, dd, *J*= 5.3, 10.8 Hz, H-9′a), 3.77 (3H, s, Ar-OCH_3), 3.77 (3H, s, Ar-OCH_3′), 3.78 (3H, s, Ar-OCH_3), 3.79 (3H, s, Ar-OCH_3′), 6.59 (1H, d, *J* = 1.9 Hz, H-2), 6.61 (1H, d, *J* = 1.9 Hz, H-2′), 6.62 (1H, dd, *J* = 1.9, 8.0 Hz, H-6), 6.64 (1H, d, *J* = 1.9, 8.0 Hz, H-6′), 6.69 (1H, *J* = 8.0 Hz, H-5), 6.71 (1H, d, *J* = 8.0 Hz, H-5′).
^{13}C NMR (151 MHz, $CDCl_3$, 25 °C) d_{13} 32.6 (C-8), 32.9 (C-7), 37.4 (C-7′), 42.1 (C-8′), 55.9 (Ar-OCH_3) 55.9 (Ar-OCH_3′), 56.0 (Ar-OCH_3) 56.0 (Ar-OCH_3′), 65.0 (C-9′), 111.2 (C-5), 111.3 (C-5′), 111.7 (C-2), 112.4 (C-2′), 120.2 (C-6), 121.2 (C-6′), 133.2 (C-1′), 135.1 (C-1), 147.3 (C-4), 147.4 (C-4′), 148.9 (C-3, C-3′).

[1] Willför, S.; Hemming, J.; Reunanen, M, Eckerman, C.; Holmbom, B. Lignans and lipophilic extractives in Norway spruce knots and stemwood. *Holzforschung* **2003,** *57*, 27–36

[2] Eklund, P.; Riska, A.; Sjöholm, R. Synthesis of R-(−)-Imperanene from the Natural Lignan Hydroxymatairesinol *J. Org. Chem*. **2002**, *67*, 7544–7546. DOI: 10.1021/jo025985c

^{1}H- and ^{13}C-NMR spectra of compounds 38-50

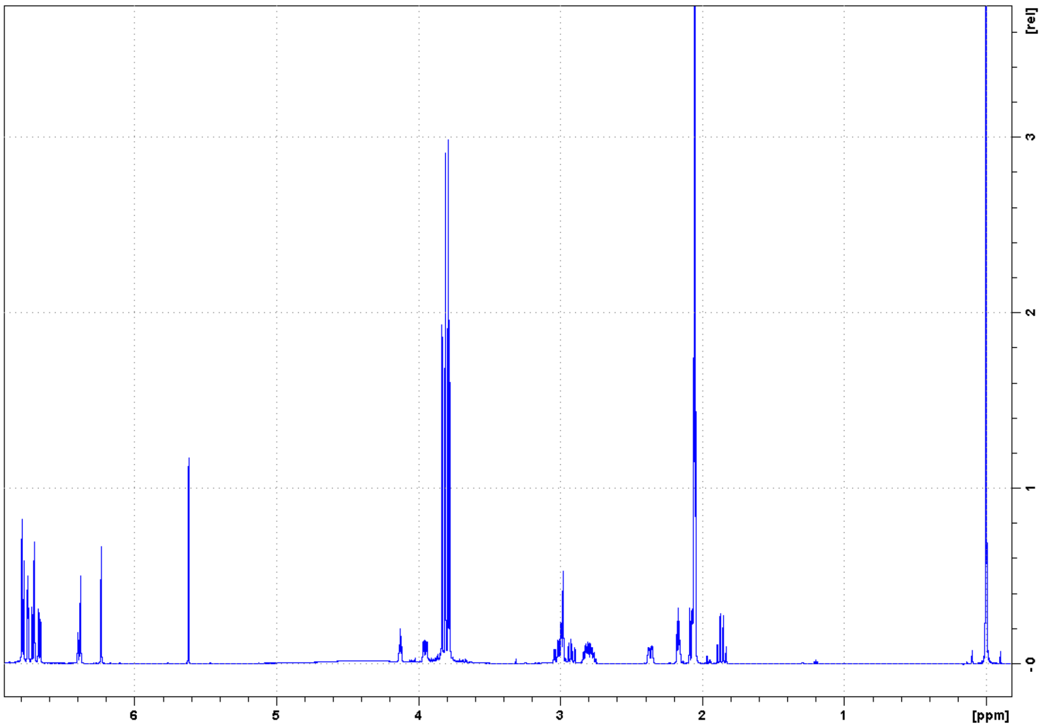

^{1}H-

^{1}H-NMR (600 MHz, $(CD_3)_2CO$) spectrum of **38** (two diastereomers).

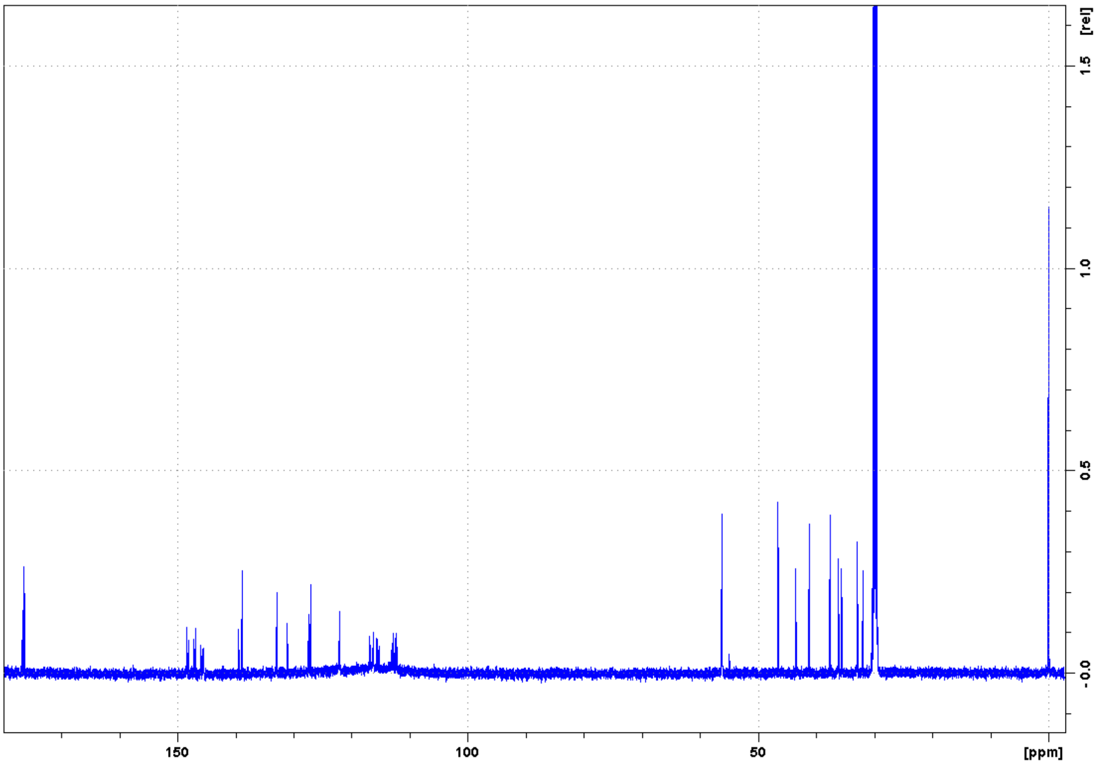

^{13}C-NMR (151 MHz, $(CD_3)_2CO$) spectrum of **38** (two diastereomers).

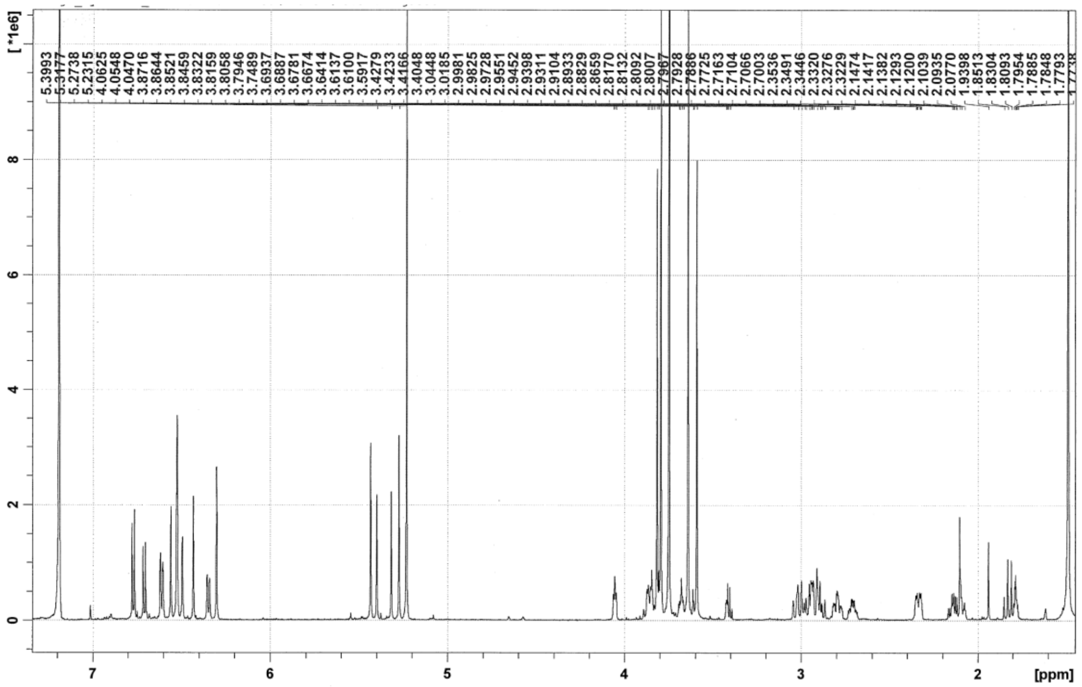

^{1}H-NMR (600 MHz, $CDCl_3$) spectrum of **39** (two diastereomers).

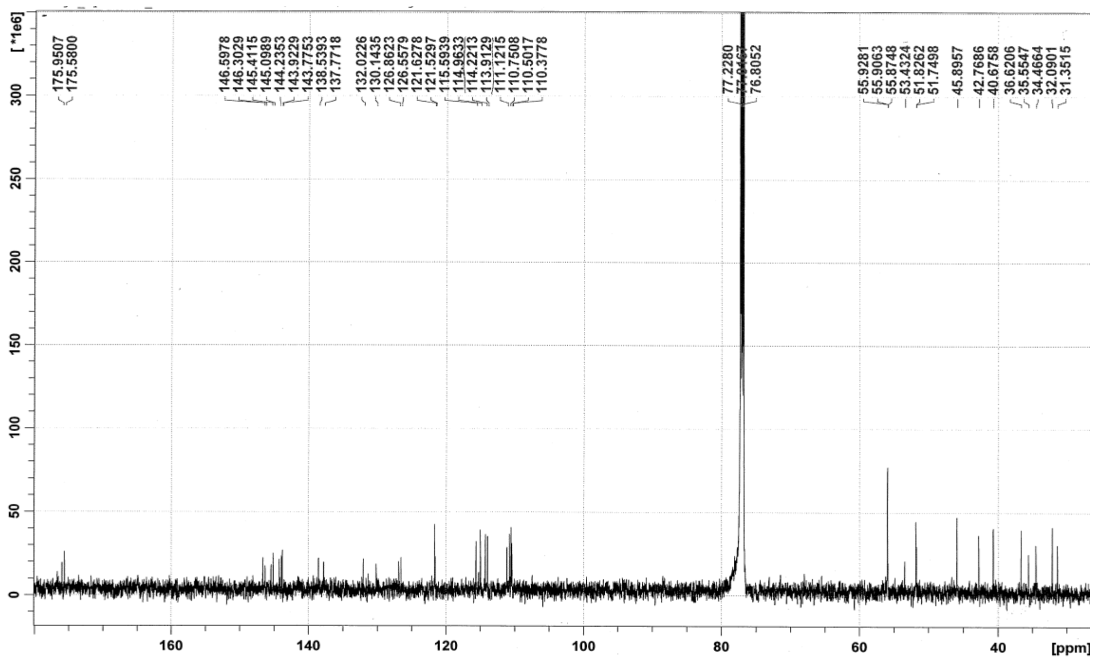

^{13}C-NMR (151 MHz, $CDCl_3$) spectrum of **39** (two diastereomers).

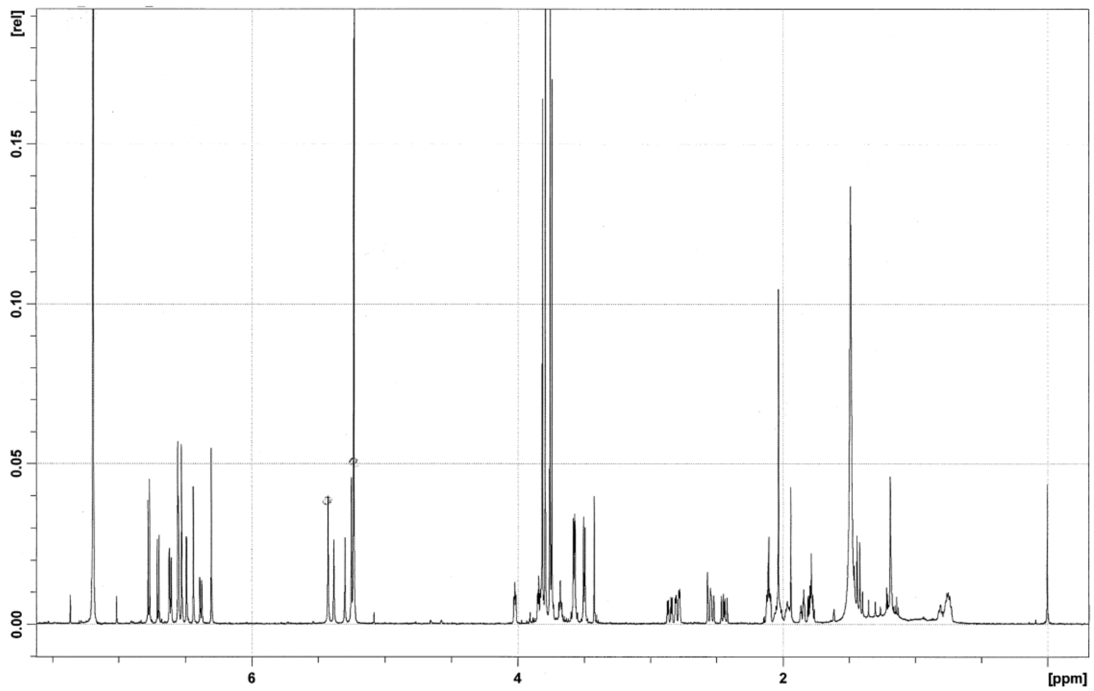

^{1}H-NMR (600 MHz, $CDCl_3$) spectrum of **40** (two diastereomers).

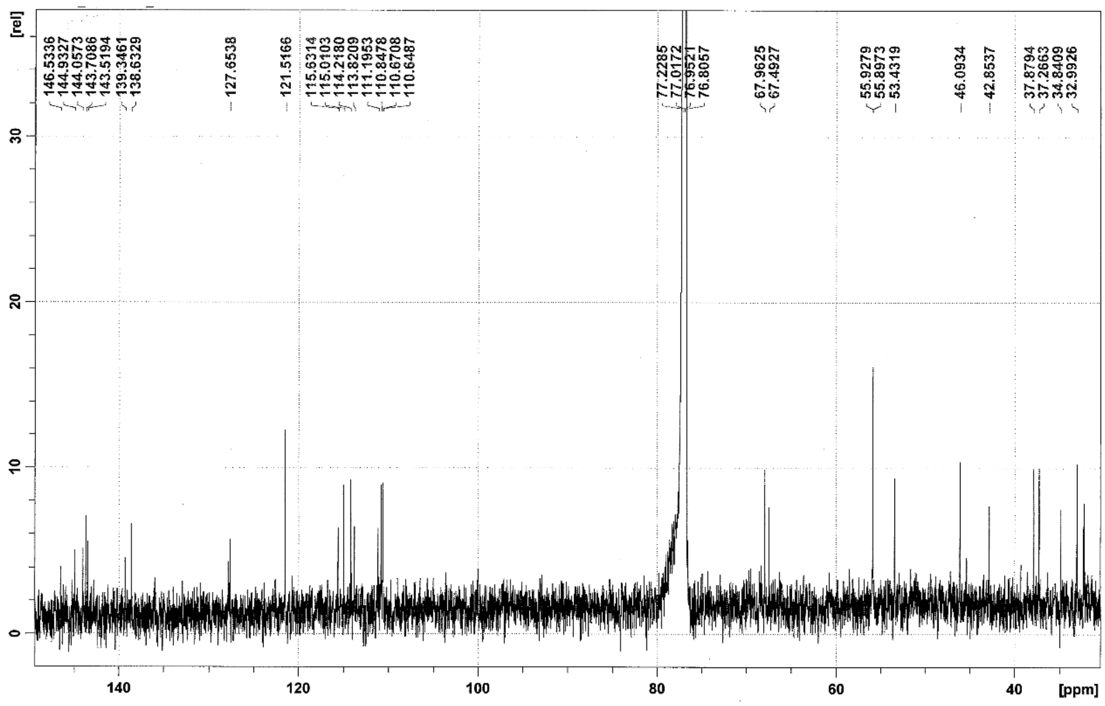

^{13}C-NMR (151 MHz, $CDCl_3$) spectrum of **40** (two diastereomers).

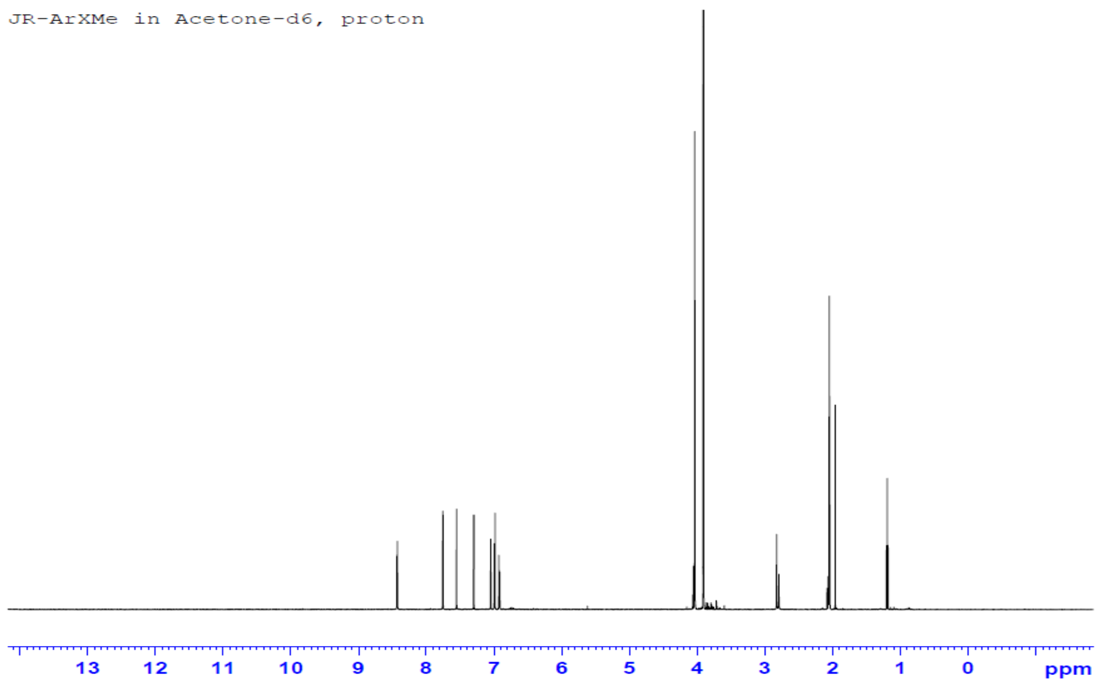

^{1}H-NMR (600 MHz, $(CD_3)_2CO$) spectrum of **41**.

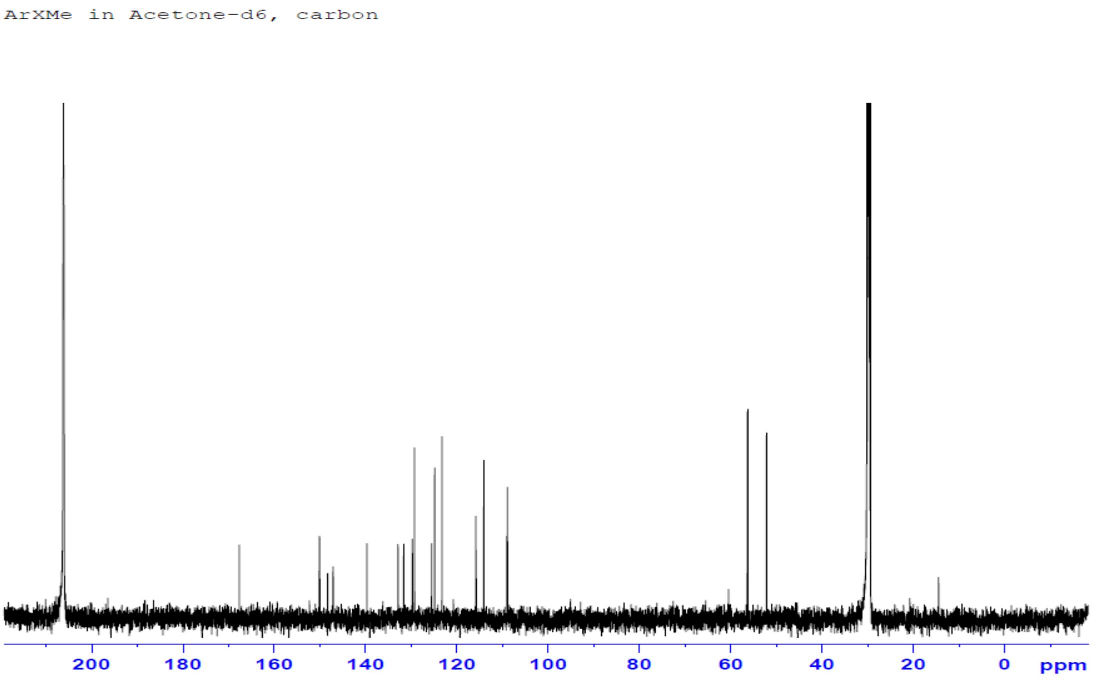

^{13}C-NMR (151 MHz, $(CD_3)_2CO$) spectrum of **41.**

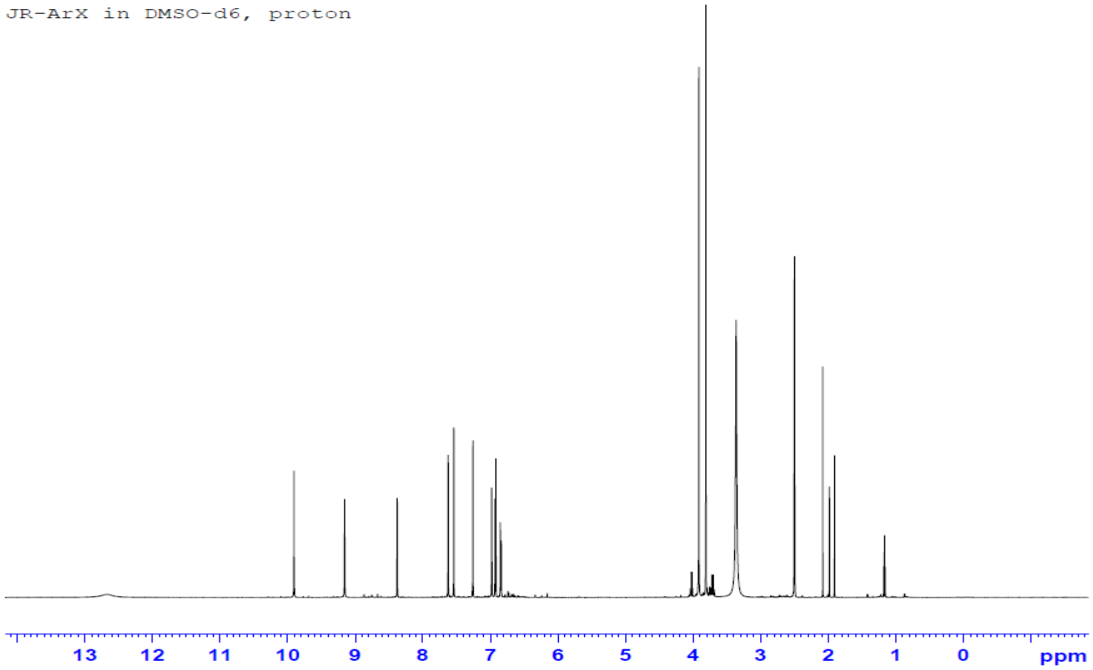

^{1}H-NMR (600 MHz, DMSO-d_6) spectrum of **42**.

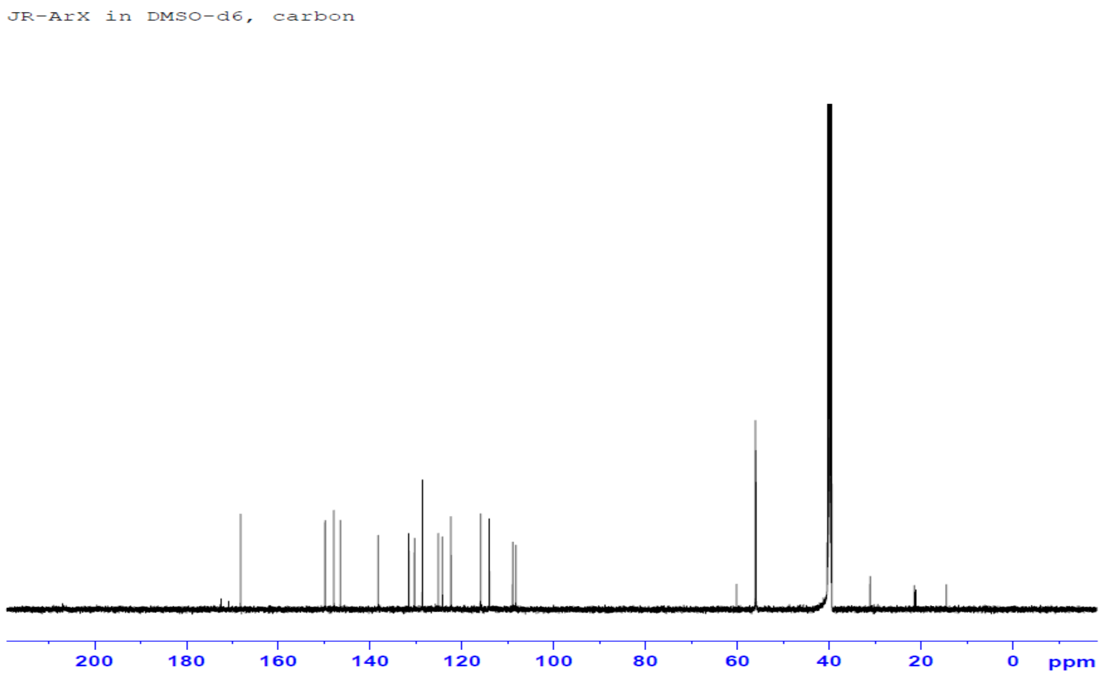

^{13}C-NMR (151 MHz, DMSO-d$_6$) spectrum of **42**.

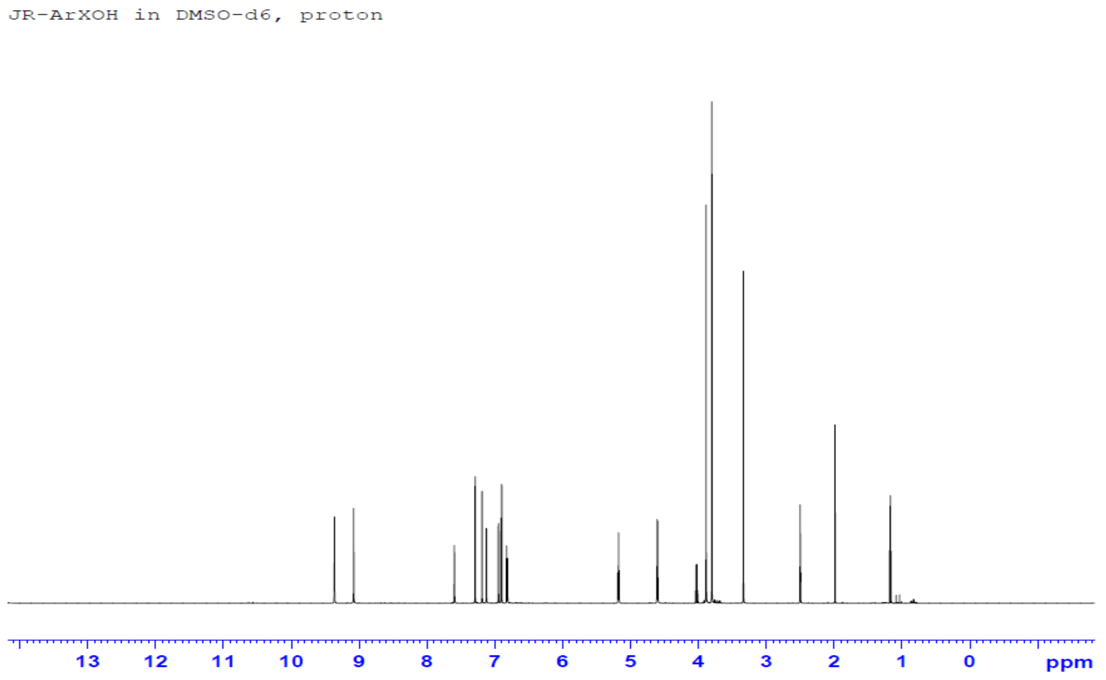

^{1}H-NMR (600 MHz, DMSO-d$_6$) spectrum of **43**.

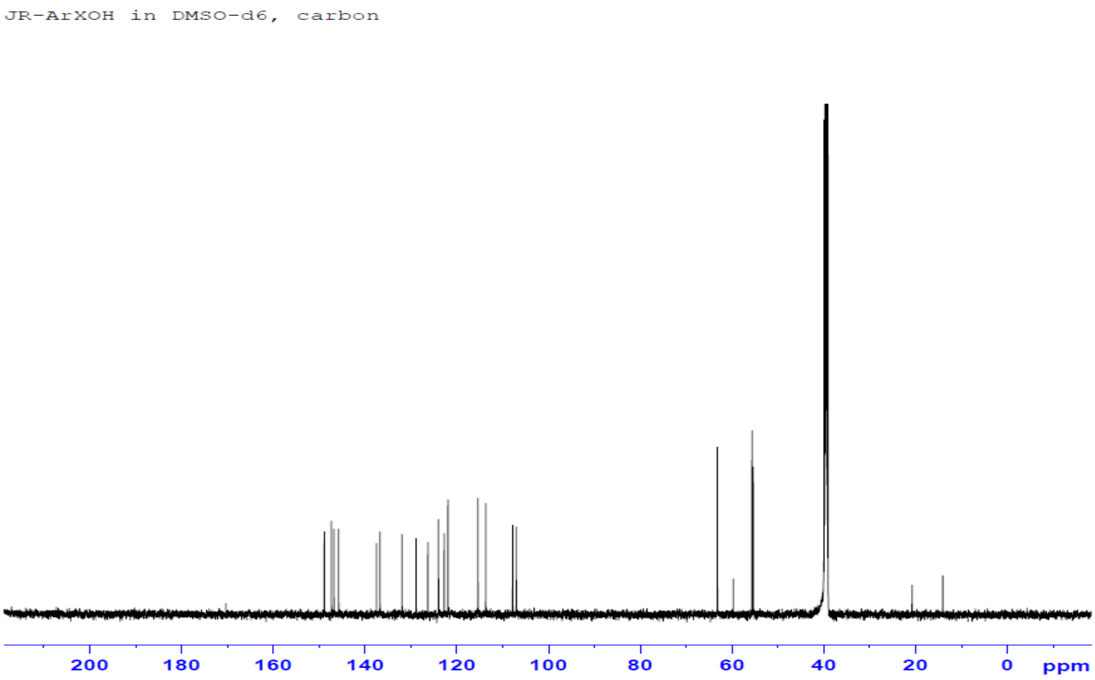

^{13}C-NMR (151 MHz, DMSO-d$_6$) spectrum of **43**.

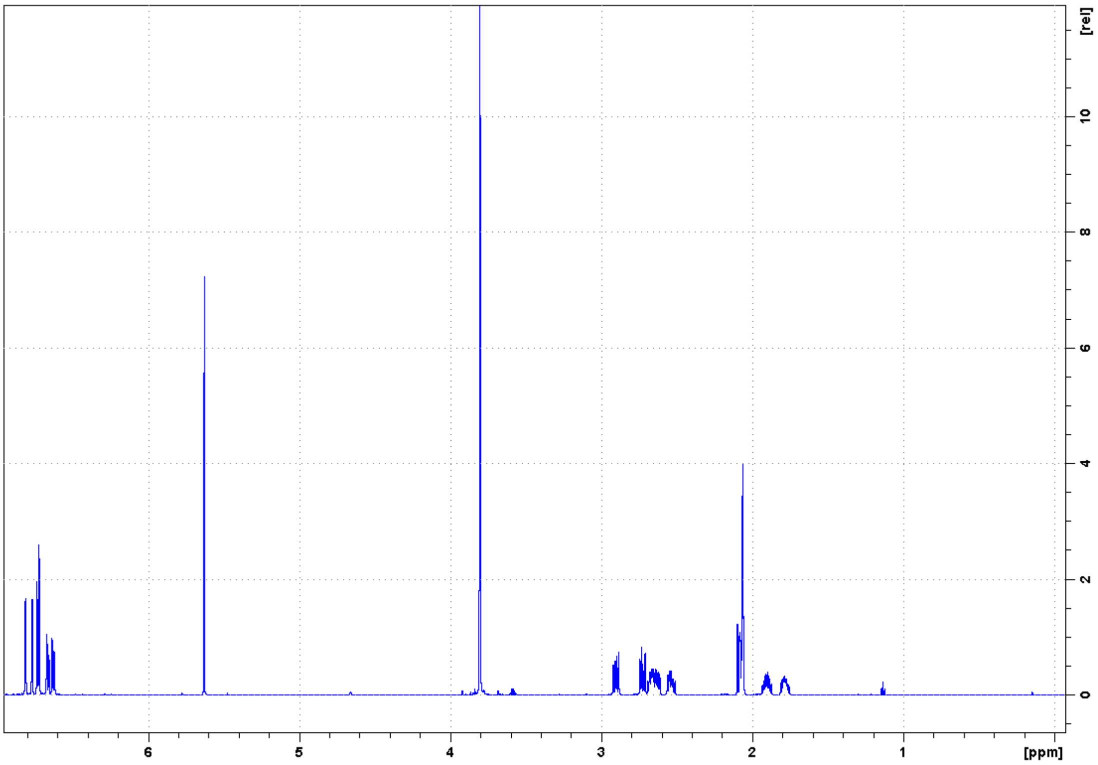

^{1}H-NMR (600 MHz, $(CD_3)_2CO$) spectrum of **44**.

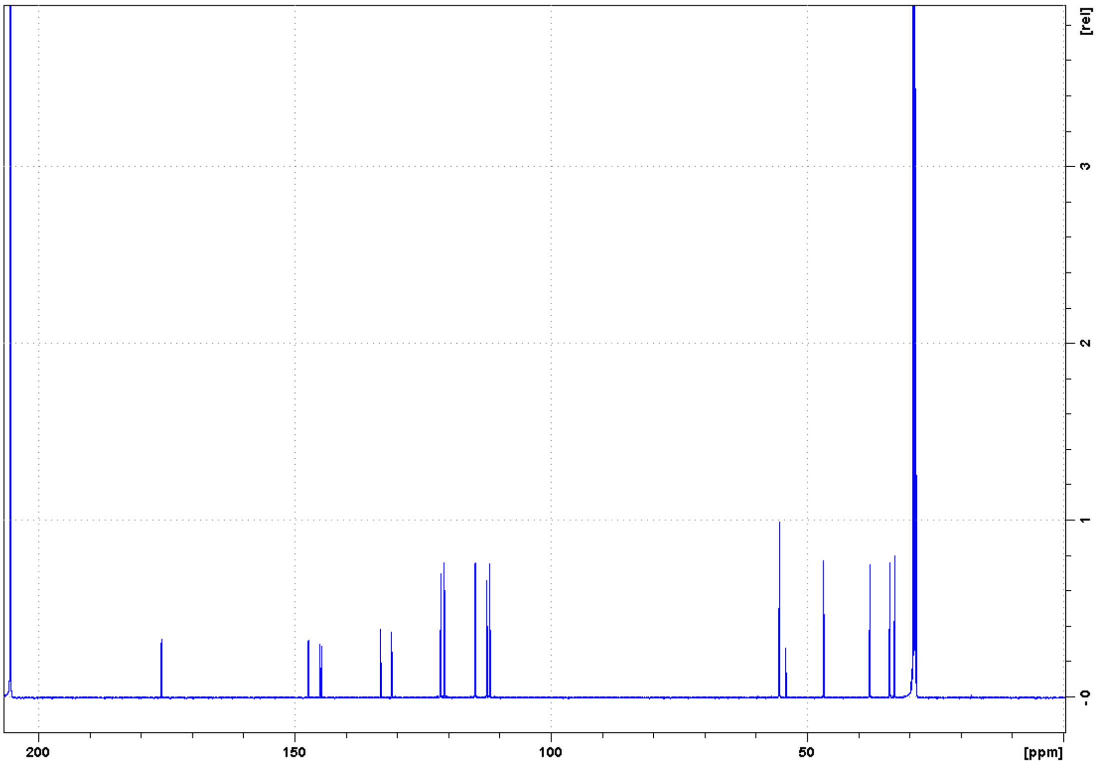

^{13}C-NMR (151 MHz, $(CD_3)_2CO$) spectrum of **44**.

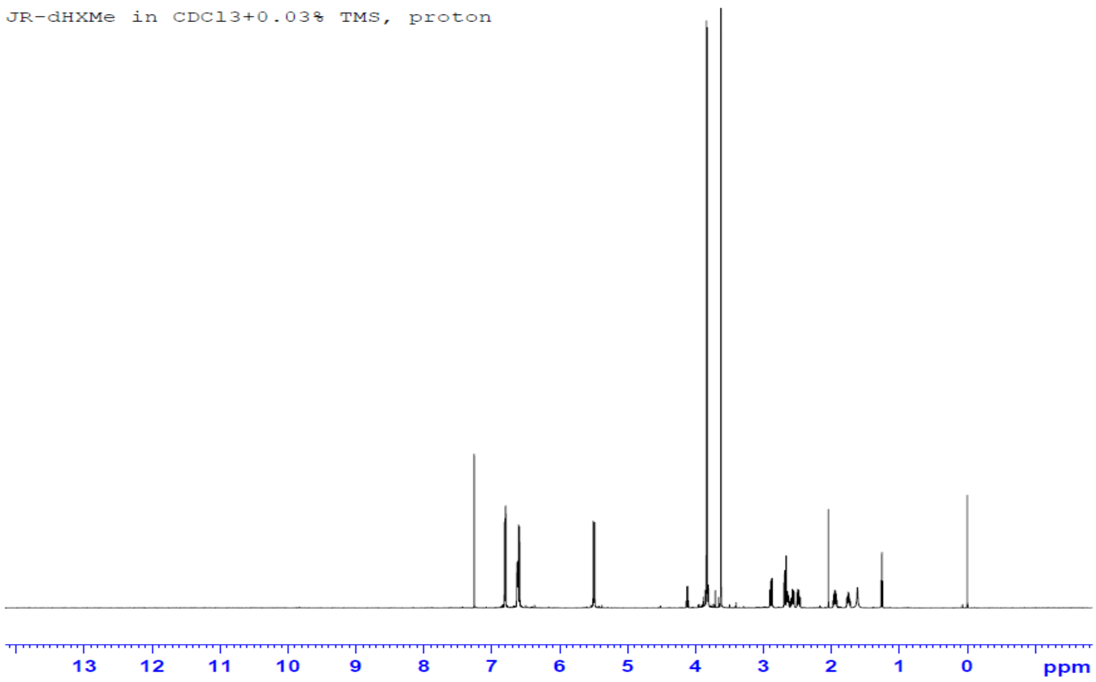

^{1}H-NMR (600 MHz, $CDCl_3$) spectrum of **45**.

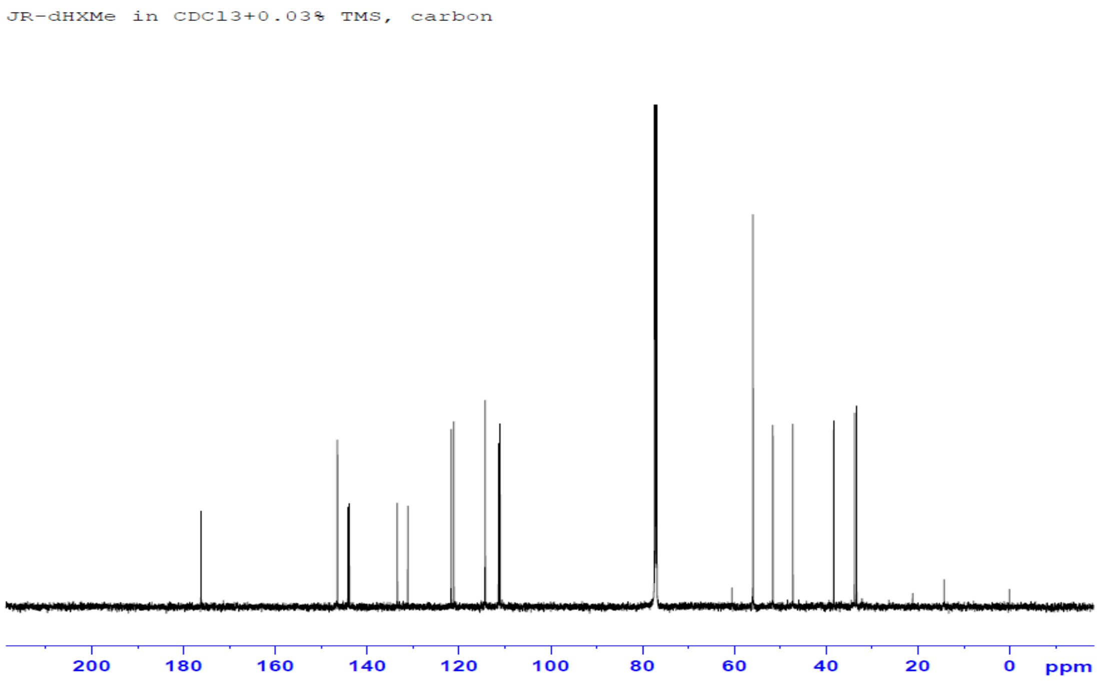

^{13}C-NMR (151 MHz, $CDCl_3$) spectrum of **45**.

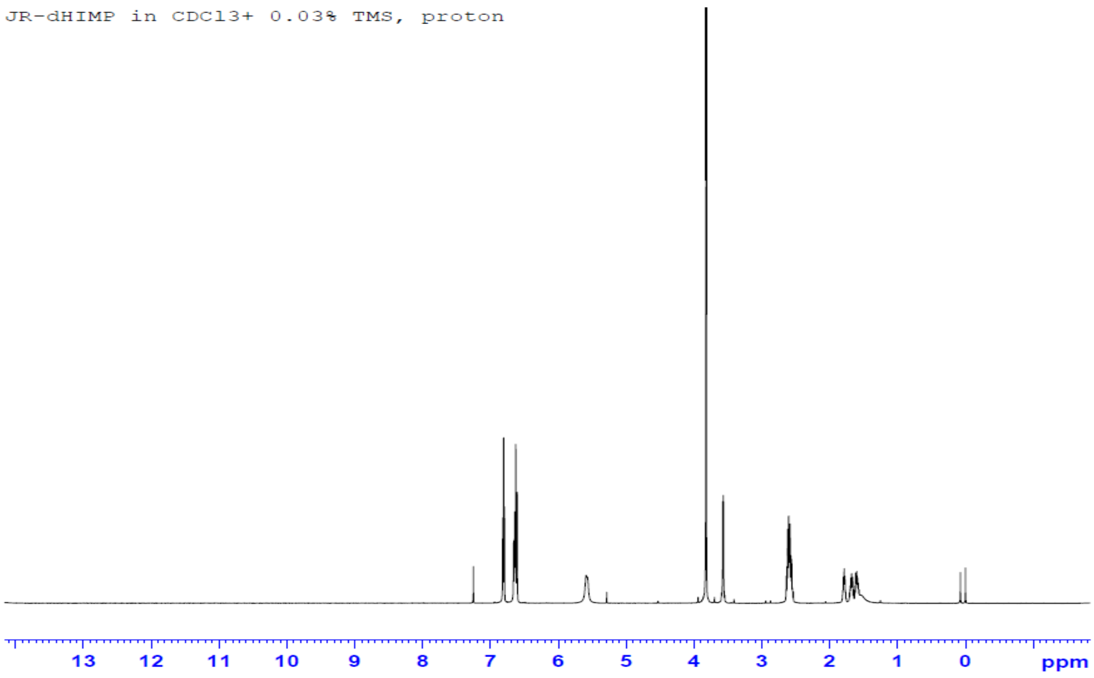

^{1}H-NMR (600 MHz, $CDCl_3$) spectrum of **46**.

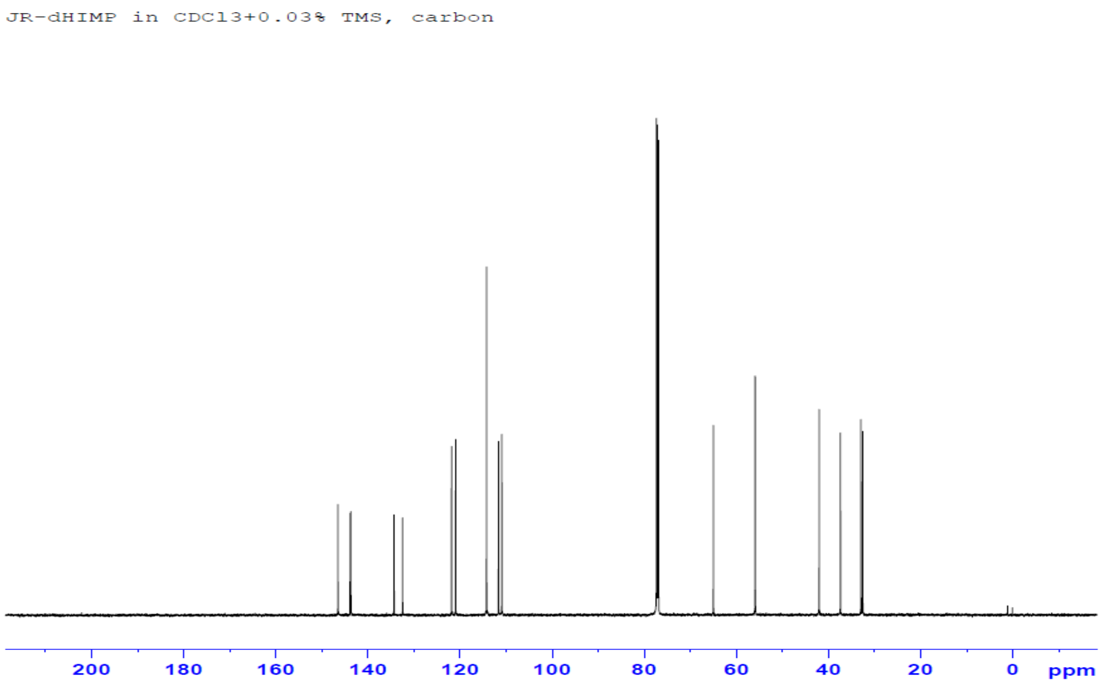

^{13}C-NMR (151 MHz, $CDCl_3$) spectrum of **46**.

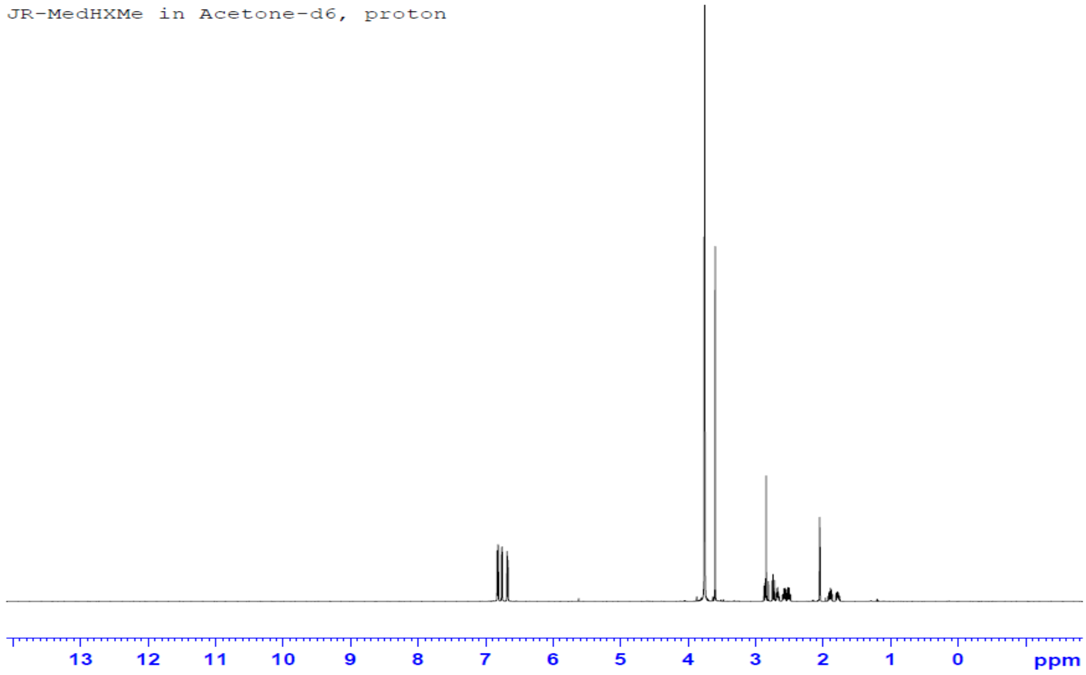

^{1}H-NMR (600 MHz, $(CD_3)_2CO$) spectrum of **48**.

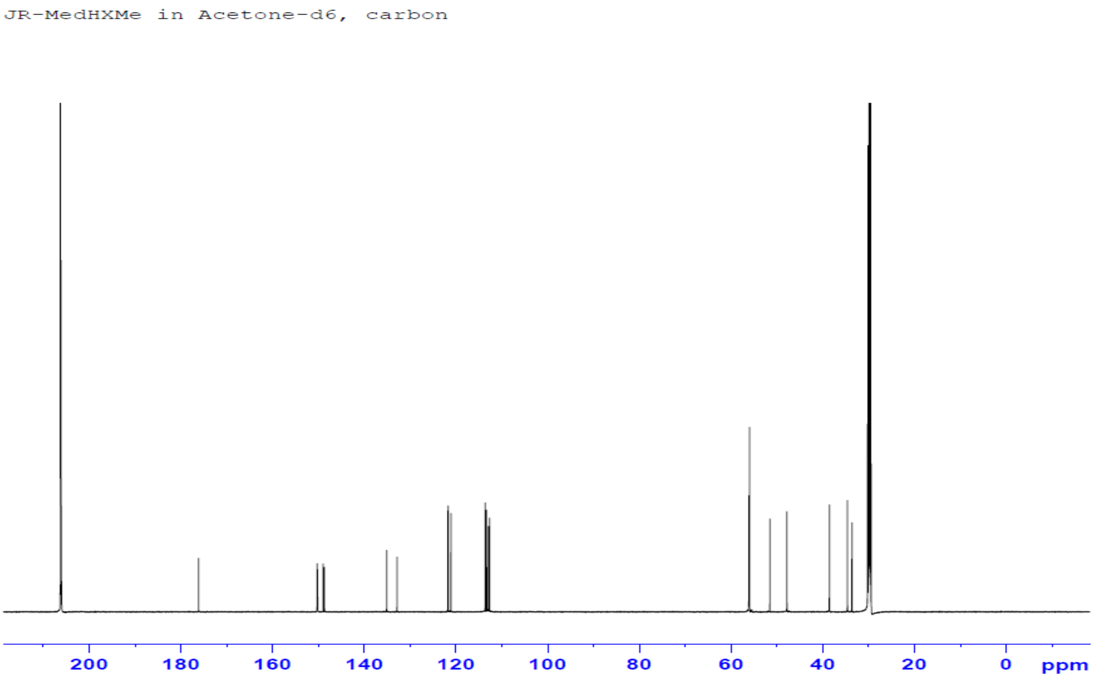

^{13}C-NMR (151 MHz, $(CD_3)_2CO$) spectrum of **48**.

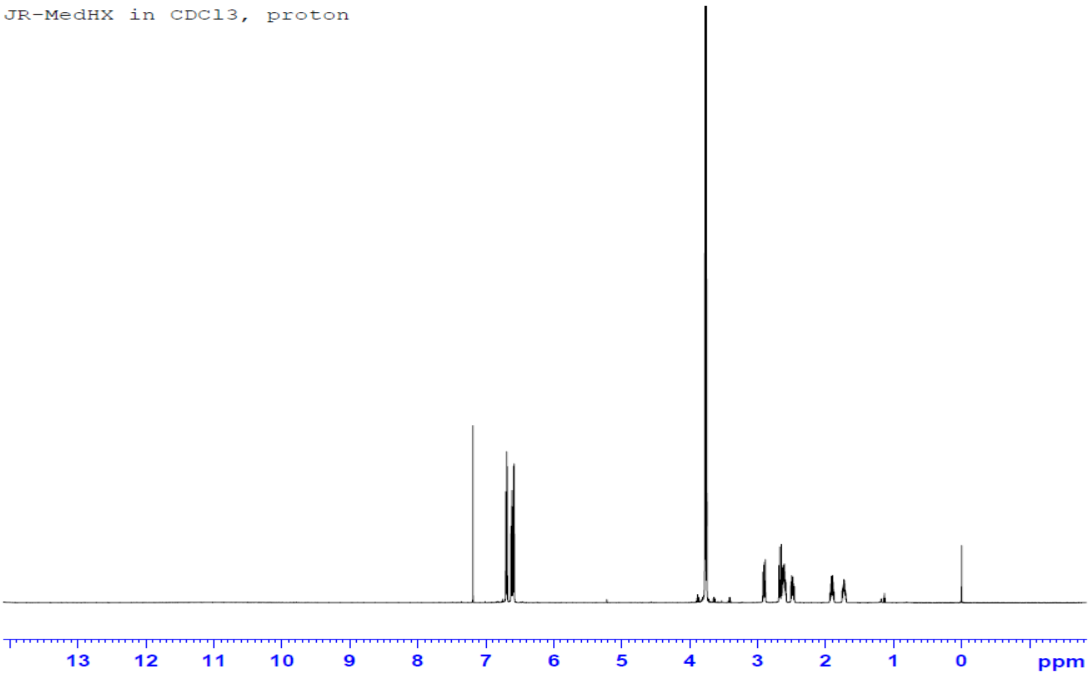

^{1}H-NMR (600 MHz, $CDCl_3$) spectrum of **47**.

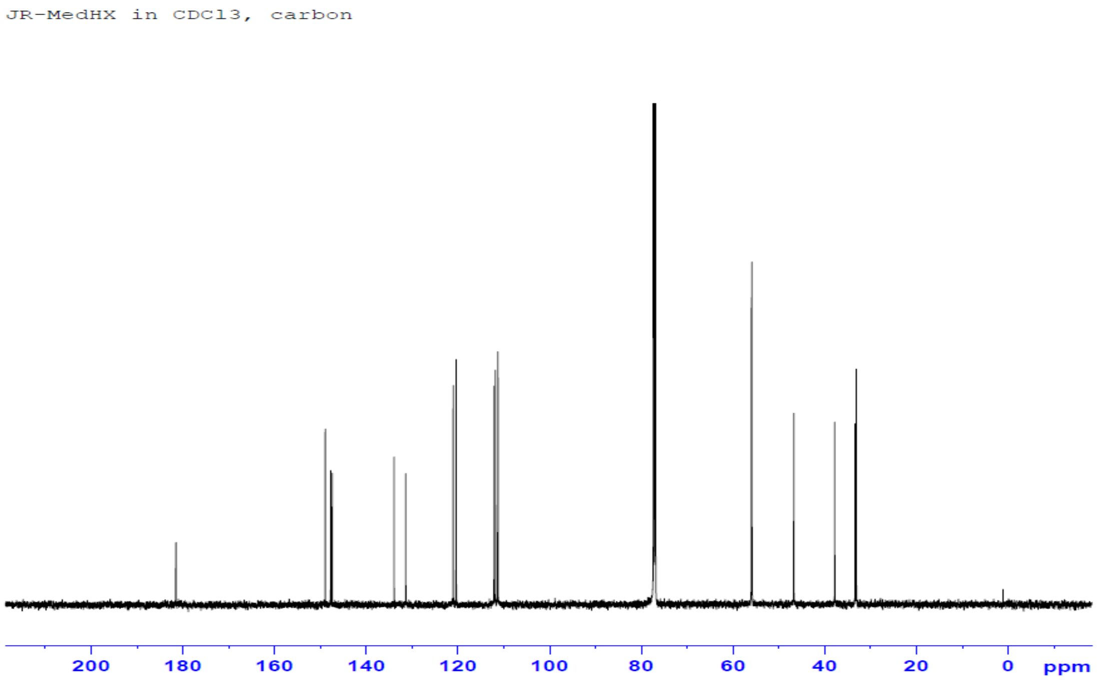

^{13}C-NMR (151 MHz, $CDCl_3$) spectrum of **47**.

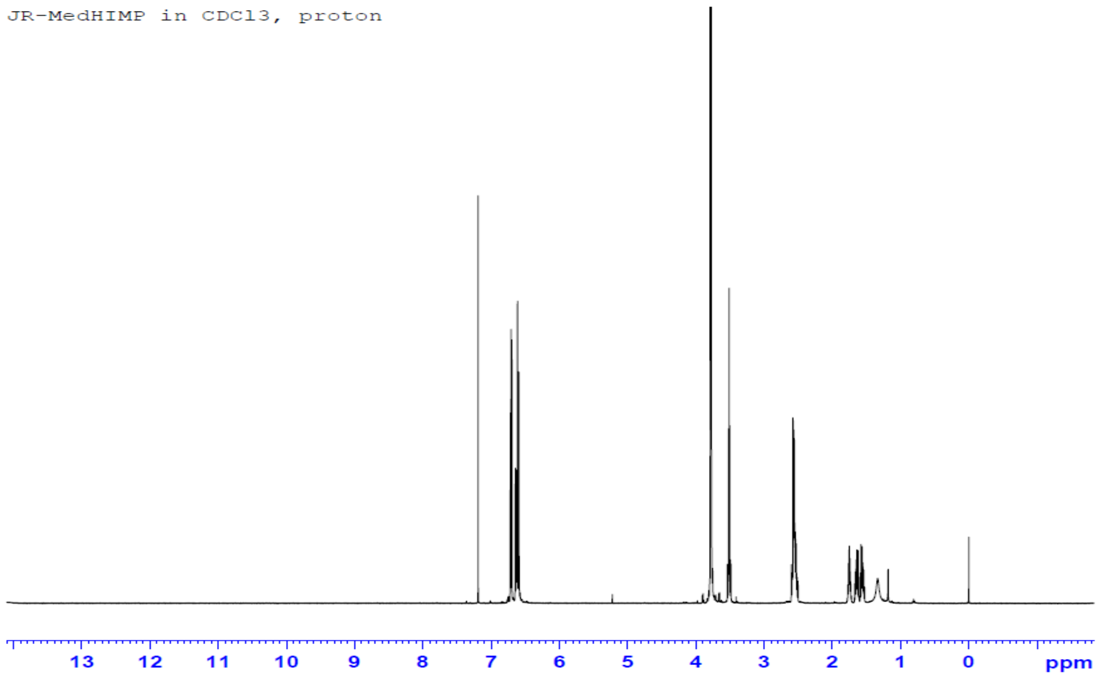

^{1}H-NMR (600 MHz, $CDCl_3$) spectrum of **49**.

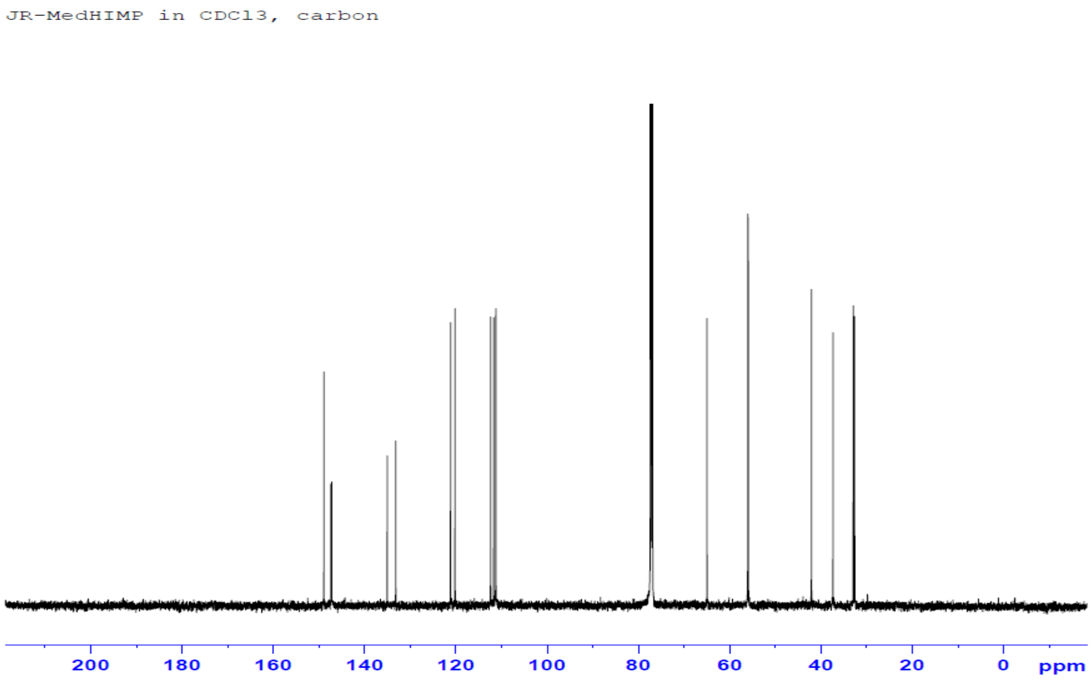

^{13}C-NMR (151 MHz, $CDCl_3$) spectrum of **49**.

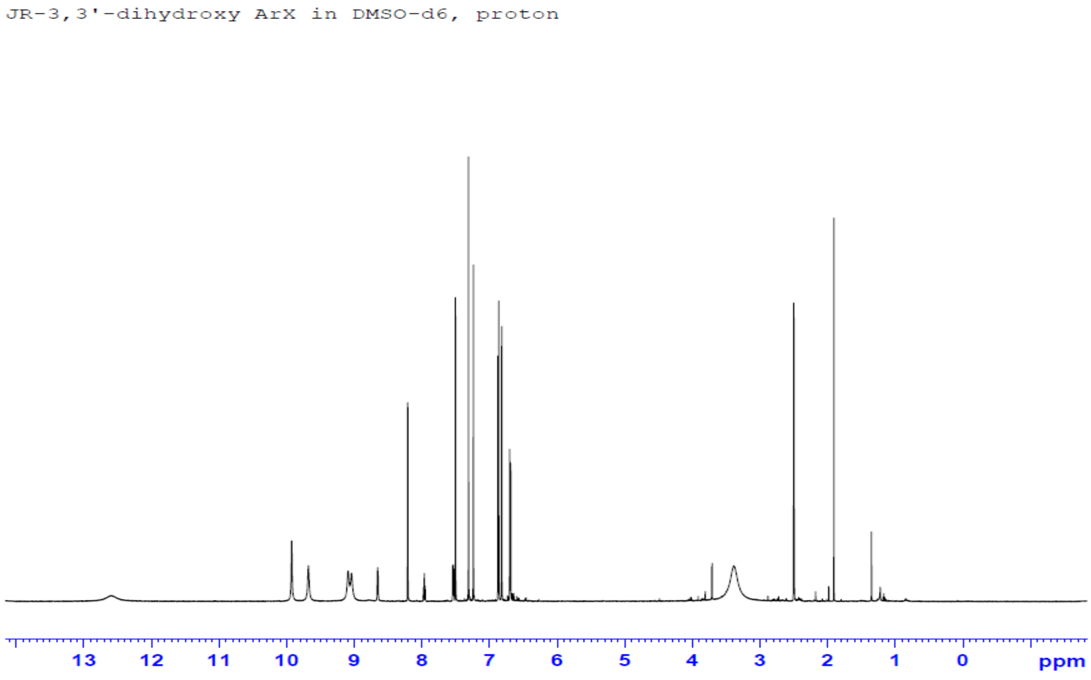

^{1}H-NMR (600 MHz, DMSO-d_6) spectrum of **50**.

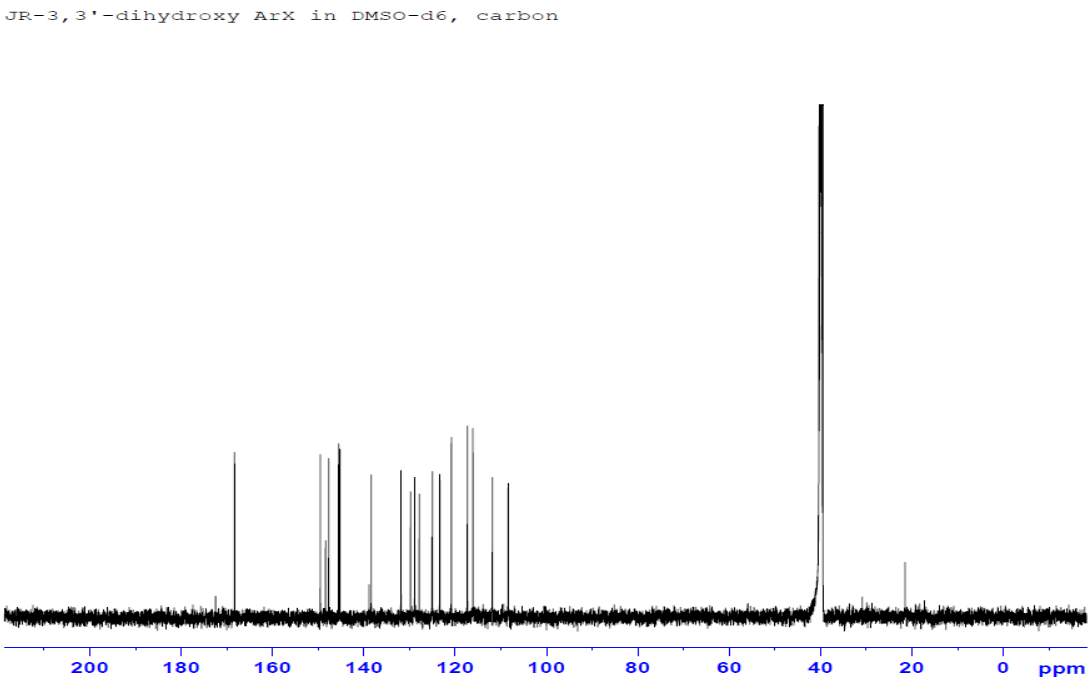

^{13}C-NMR (151 MHz, DMSO-d_6) spectrum of **50**.
